# Expansion, functional diversification, and gene fusion events in the Ato protein family

**DOI:** 10.1016/j.isci.2026.116467

**Published:** 2026-06-22

**Authors:** Faezeh Ghasemi, Patrícia Ataíde, Cláudia Barata-Antunes, Yiannis Pyrris, João Alves, Vitor Fernandes, Rosana Alves, Alexandra Gomes-Gonçalves, Margarida Casal, Wouter Van Genechten, Jana Nysten, Alistair J.P. Brown, Patrick Van Dijck, Alexandros A. Pittis, George Diallinas, Isabel Soares-Silva, Sandra Paiva

**Affiliations:** 1 Centre of Molecular and Environmental Biology, Department of Biology, University of Minho, Braga, Portugal; 2 Institute of Science and Innovation for Bio-Sustainability (IB-S), University of Minho, Braga, Portugal; 3 Institute of Molecular Biology and Biotechnology of the Foundation for Research and Technology, 70013 Heraklion, Greece; 4 Department of Biology, National and Kapodistrian University of Athens, Panepistimiopolis 15784 Athens, Greece; 5 Laboratory of Molecular Cell Biology, Institute of Botany and Microbiology, K.U. Leuven, Leuven, Belgium; 6 MRC Centre for Medical Mycology, University of Exeter, Exeter, England, UK; 7 KU Leuven One Health Institute, Leuven, Belgium

**Keywords:** Molecular modeling, Structural biology, Protein structure aspects, Membrane Dynamics, Transporter Biology, Structure-function analysis

## Abstract

The human commensal, *Candida albicans,* adapts to glucose-limited niches by utilizing alternative carbon sources such as carboxylates. *Saccharomyces cerevisiae* can assimilate monocarboxylates via Ato1 (Ady2), a member of the Acetate Uptake Transporter (AceTr) family. In *C. albicans,* this Ato family has expanded significantly to 10 members (Ato1–Ato10), most with unknown functions. Therefore, we investigated the roles of *C. albicans* Ato proteins in carboxylate utilization. Functional diversification of *C. albicans* Atos (CaAtos), suggested by *in silico* analyses of their AceTr motifs, pore radii, and substrate-binding sites, was confirmed by experimental dissection of their carboxylate transport capacities, revealing CaAto1 as the major acetate transporter, driven by the proton motive force. CaAto1-3 and CaAto6 showed carboxylate-dependent expression and plasma membrane localization. Furthermore, *CaATO1* deletion resulted in endoplasmic reticulum (ER) retention of CaAto2 and loss of CaAto3 expression, indicating a central regulatory role for CaAto1. Our analyses reveal further evolutionary diversification of the Ato family in vertebrates.

## Highlights

• The 10-member CaAto family displays distinct binding sites and pore architectures
• Ato1 is the main acetate transporter in *Candida albicans*, operating via H^+^ symport
• Carboxylates induce plasma membrane localization of Ato1, 2, 3, and 6
• Ato1 influences Ato2 membrane localization and is required for Ato3 expression

## Graphical abstract

**Figure:**
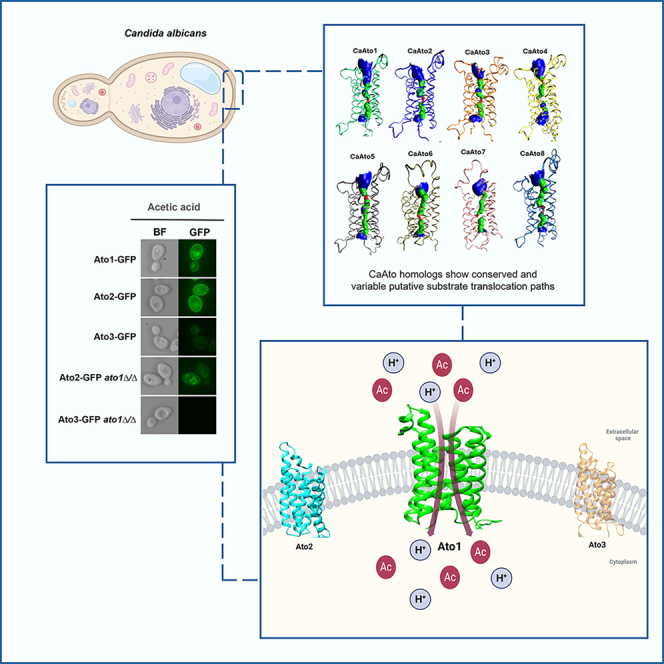

## Introduction

Opportunistic fungal pathogens of humans include several *Candida* species that have become serious threats to global public health.^1^^,^^2^^,^^3^^,^^4^ These fungi can cause a broad spectrum of diseases, ranging from superficial infections of the vaginal and oral mucosa to life-threatening systemic infections.^5^^,^^6^ The genus *Candida* comprises 200 species, with approximately 10% identified as human pathogens.^7^ The most clinically significant species include *Candida albicans*, *Candida glabrata* (*Nakaseomyces glabratus*), *Candida auris* (*Candidozyma auris*), *Candida tropicalis*, *Candida parapsilosis*, and *Candida krusei* (*Pichia kudriavzevii*).^8^^,^^9^^,^^10^^,^^11^^,^^12^ *C. albicans,* a commensal microorganism, exhibits the capacity to colonize diverse host niches, including the skin, oral cavity, gastrointestinal tract, and urogenital tract.^13^^,^^14^^,^^15^ Considerable metabolic flexibility underlies this adaptability.^16^^,^^17^

Although sugars are the preferred carbon sources for *C. albicans*,^18^ this pathogen can rapidly adapt to glucose-limited environments, such as the gastrointestinal tract.^19^^,^^20^ In these niches, *C. albicans* cells can utilize alternative carbon sources, including carboxylates (lactate, acetate, succinate, butyrate, or propionate), amino acids, and N-acetylglucosamine, which are produced by host cells or the resident microbiota.^16^^,^^20^^,^^21^^,^^22^^,^^23^ Carboxylic acids exist in two forms depending on their pKa and the ambient pH. When the environmental pH is lower than the pKa, the protonated (undissociated) form of the acids can cross the plasma membrane (PM) by passive diffusion. Conversely, when the pH exceeds the acid’s pKa, the acid is predominantly in its anionic (dissociated) form, which requires protein-mediated systems to cross the PM.^24^^,^^25^

The Acetate Uptake Transporter (AceTr) family (TCDB 2.A.96, Pfam: PF01184) is evolutionarily conserved across bacteria, archaea, and fungi.^26^ A majority of fungal genomes (97%) harbor members of this family, suggesting crucial roles in fungal growth and development.^27^^,^^28^ Key transporters of the AceTr family include Gpr1 in *Yarrowia lipolytica*,^29^ Ady2/Ato1 in *Saccharomyces cerevisiae*,^30^ AcpA in *Aspergillus nidulans*,^28^ and SatP in bacteria.^31^ AceTr family members carry six transmembrane segments (TMSs). The conserved signature motif NPAPLGL(M/S) at the beginning of the first TMS plays a critical role in substrate uptake.^26^^,^^32^ The only elucidated crystal structures among AceTr family members are those of SatP from *Escherichia coli* (EcSatP) and *Citrobacter koseri* (CkSatP).^33^^,^^34^ Structural analysis of SatP has confirmed six transmembrane helices, four substrate-binding sites, and a constriction site located in the center of the protein pore. This constriction site is formed by three hydrophobic residues, phenylalanine (F), tyrosine (Y), and leucine (L), known as the FLY motif (structural element), which is essential in substrate selectivity and specificity.^32^^,^^33^

In *E*. *coli*, EcSatP facilitates the transport of monocarboxylates (acetate and lactate) and dicarboxylate (succinate).^31^^,^^32^^,^^33^ In *S. cerevisiae*, Ato1 (Ady2) is identified as an acetate permease with an additional capability to transport other monocarboxylates, including propionate, formate, and lactate.^26^^,^^30^ Indeed, the *ScATO1* gene is required for protein-mediated acetate import when *S. cerevisiae* cells are grown in acetate at pH 6.0.^30^ Ato1 has two paralogs in *S. cerevisiae*: Ato2 (Fun34) and Ato3. The expression of these three proteins is induced significantly under carbon-limiting conditions and following entry to stationary phase after growth on glucose.^35^^,^^36^ Palková et al., 2002,^37^ reported that the ScAto1-3 is required for extracellular alkalinization during colony development, a process associated with ammonium release.

Homologs of *ScATO1* have been found in *Candida* species. Indeed, 12 of the most medically relevant *Candida* spp. contain at least two *ScATO1* homologs, and the majority have five or more. The expansion of *ATO* gene families in *Candida* pathogens reinforces the idea that they play important roles in fungal adaptation and probably contribute to virulence.^8^ *C. albicans* has a large AceTr family that includes 10 *ATO* (Acetate Transporter Ortholog) genes (*ATO1*, *ATO2*, *ATO3*, *ATO4*, *ATO5*, *ATO6*, *ATO7*, *ATO8*, *ATO9*, and *ATO10*).^8^^,^^22^ The functions of their respective proteins remain largely unknown. *CaATO9* and *CaATO10* are probably pseudogenes, originating from a single gene that was disrupted by insertion of a transposable element.^8^ Previous studies have shown that the neutralization of the acidic phagolysosome induces the differentiation of *C. albicans* yeast cells to form filamentous hyphae, thereby facilitating their escape from macrophages via immune cell rupture.^38^ Notably, mutations in certain *C. albicans ATO* genes diminish this alkalinization process, which correlates with defects in filamentation and reduced survival within host immune cells.^22^^,^^38^ Despite these functional links, the contribution of *C. albicans* Ato (CaAto) proteins to systemic virulence has not yet been demonstrated.^39^^,^^40^ We recently showed that deletion of the entire *ATO* gene family significantly reduces *C. albicans* colonization of the murine gut, particularly after antibiotic elicited bacterial disruption, indicating that *ATOs* are important for long-term persistence in the gut.^41^

In this study, we focused on the phylogenetic analyses of AceTr family members, the *in silico* structural characterization of Ato proteins in divergent *Candida* species (*C. albicans, C. glabrata*, and *C. auris*), and the cellular and functional expression of CaAto proteins. Our analyses reveal the conservation of significant AceTr-specific motifs across the mentioned species alongside the presence of distinct pore radii in the respective Ato proteins, suggestive of distinct transport activities and specificities. Additionally, we demonstrate that CaAto1, CaAto2, CaAto3, and CaAto6 are differentially expressed in *C. albicans*, with CaAto1 playing a key role in acetate transport and in the regulation of the expression and localization of other Ato family members. We define CaAto1 as a H^+^-symporter that functions as a high-affinity acetate transporter while exhibiting low-affinity transport for other carboxylates. Finally, our phylogenetic analyses uncover a previously unknown ATO-related family in vertebrates that contains Ato-like transport domains. Unexpectedly, these domains are fused to an Sua5/YciO/YrdC module implicated in the formation of threonyl-carbamoyl-adenosine during tRNA modification.

## Results

### CaAto members possess conserved NPAPLGL, FLY, and SY[F]GFW motifs

**Figure 1:**
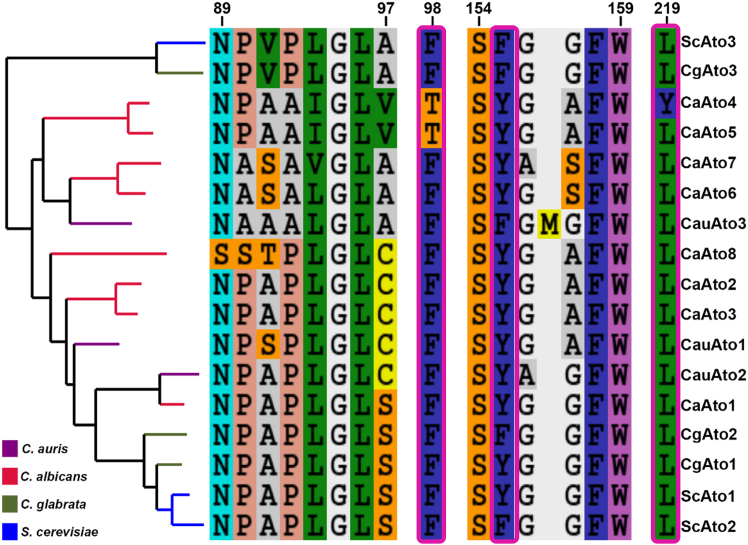
Phylogenetic analysis and conserved residues of Ato homologous proteins from *C. albicans* (Ato1-8), *C. glabrata* (Ato1-3), *C. auris* (Ato1-3), and *S. cerevisiae* (Ato1-3) The signature motif, N-P-A-P-L-G-L, of the AceTr family; the narrowest hydrophobic constriction region (FLY; highlighted in pink); and the signature motif S-Y[F]-G-F-W are mapped alongside with the phylogenetic tree. Full alignment is available in the supplementary material (Figure S2).

Multiple sequence alignment of AceTr members from *S. cerevisiae*, *C. albicans*, *C. glabrata*, and *C. auris* showed that CaAto1-8, CgAto1-3, CauAto1-3, and ScAto1-3 contain common conserved regions (Figure 1; Table 1). *C. albicans* Ato9 and Ato10 were excluded from this analysis as they are likely functionally inactive.^8^ However, manually fusing CaAto9 (2 TMSs near the N terminus) and CaAto10 (4 TMSs near the C terminus) sequences does yield a structure similar to other *C. albicans* Atos (Figure S1), which may suggest that they represent fragments of one single gene. Notably, the putative functional ^88^NPAPLGL^94^ signature motif (numbering refers to CaAto-1), which is localized at the beginning of the first TMS (Figure S2), is fully conserved in CaAto1-3, CgAto1-2, CauAto2, and ScAto1-2 (Figure 1). In CaAto6-8 and CauAto3, the first Pro residue in this motif (Pro89) is substituted by Ala or Ser. In addition, in CaAto4-7 and CauAto3, the second Pro residue (Pro91) is substituted by Ala. The highly conserved residue Leu92 is substituted by isoleucine in CaAto4 and CaAto5 and valine in CaAto7. Therefore, CaAto7 and CaAto8 exhibit the highest divergence in the NPAPLGL motif in these Ato families, differing in four and three of the seven residues in this motif, respectively. These differences are likely to change the substrate affinities, specificities, and/or activities of these transporters. This prediction is based on previous mutational analyses of ScAto1, which established that amino acid substitutions in this motif impaired protein function per se, without affecting its proper localization at the PM.^42^

**Table 1:** Nomenclature of *ATO* family genes and protein accession numbers in the NCBI and AlphaFold databases for *C. albicans*, *C. glabrata*, *C. auris*, and *S. cerevisiae*

Species	Database nomenclature	Alias^8^	Accession no. in NCBI	Accession no. in AlphaFold database
*C. albicans*	*FRP3*	*ATO1*	NCBI: XP_710295.1	AlphaFold: AF-A0A1D8PHP8-F1
	*ATO1*	*ATO2*	NCBI: XP_710650.1	AlphaFold: AF-A0A1D8PJ22-F1
	*ATO2*	*ATO3*	NCBI: XP_718515.2	AlphaFold: AF-A0A1D8PJ42-F1
	*ATO6*	*ATO4*	NCBI: XP_714701.1	AlphaFold: AF-A0A1D8PKA0-F1
	*ATO5*	*ATO5*	NCBI: XP_714703.1	AlphaFold: AF-A0A1D8PKD7-F1
	*FRP6*	*ATO6*	NCBI: XP_716748.1	AlphaFold: AF-Q5A4K0-F1
	*FRP5*	*ATO7*	NCBI: XP_716747.2	AlphaFold: AF-A0A1D8PPM3-F1
	*ATO7*	*ATO8*	NCBI: XP_019330752.1	AlphaFold: AF-A0A1D8PGM7-F1
	*ATO9*	*ATO10*	NCBI: XP_717951.1	AlphaFold: AF-Q5A867-F1
	*ATO10*	*ATO9*	NCBI: XP_717953.1	AlphaFold: AF-Q5A865-F1
*C. glabrata*	*GVI51_M03377*	*ATO1*	NCBI: XP_449497.1	AlphaFold: AF-Q6FJU7-F1
	*CAGL0L07766g*/*ADY2*	*ATO2*	NCBI: XP_449115.1	AlphaFold: AF-Q6FKX9-F1
	*ATO3*	–	NCBI: XP_444907.1	AlphaFold: AF-Q6FYA8-F1
*C. auris*	*CJI97_002436*/*ATO7*	*ATO1*	NCBI: XP_028889701.1	AlphaFold: AF-A0A2H0ZYE7-F1
	*FRP3*	*ATO2*	NCBI: XP_028890140.1	AlphaFold: AF-A0A2H0ZRA6-F1
	*CJI97_003024*/*CJI96_0000785*	*ATO3*	NCBI: XP_028890274.1	AlphaFold: AF-A0A2H0ZM18-F1
*S. cerevisiae*	*ADY2*	*ATO1*	NCBI: NP_009936.1	AlphaFold: AF-P25613-F1
	*ATO2*	–	NCBI: NP_014399.3	AlphaFold: AF-C7GLQ2-F1
	*ATO3*	–	NCBI: NP_010672.1	AlphaFold: AF-Q12359-F1

Database nomenclature refers to the nomenclature used in NCBI.

Alias denotes the nomenclature recently proposed by our group.^8^ The Alias was used for the analyses performed in this study.

In ScAto1, the narrowest constriction site is formed by the FLY structural element. Previous studies have demonstrated that mutations of FLY residues in ScAto1 lead to altered protein activity and specificity, highlighted by the observation that Leu219Ala allows cells to grow on succinic acid.^32^ All analyzed *Candida* homologs possess a well-conserved phenylalanine in this FLY motif (Phe98, Figure 1), with the exception of CaAto4 and CaAto5, where it has been substituted by threonine. The substitution of a bulky hydrophobic residue by a polar amino acid might influence substrate transport through the inner pore of the transporter, as previously described for ScAto1.^32^ The Tyr residue of the FLY motif is also highly conserved, being replaced by phenylalanine, a residue with similar biochemical character, in ScAto2, ScAto3, CgAto2, CgAto3, and CauAto3 (Figure 1). Finally, the Leu residue of the FLY motif is nearly absolutely conserved, the only exception being CaAto4, where it is replaced by a Tyr. The Tyr residue of the FLY structural motif is also contained within the SY[F]GFW linear signature motif of the AceTr family, located in TMS3 (Figure S2). This motif is also highly conserved among the Ato proteins examined.

Overall, our analyses suggest that conserved evolutionary substitutions in the NPAPLGL, FLY, and SY[F]GFW motifs might have modulated the transport activities and/or substrate specificities of Ato proteins in these *Candida* pathogens.

### *C. albicans* Atos have distinct pore radii

**Figure 2:**
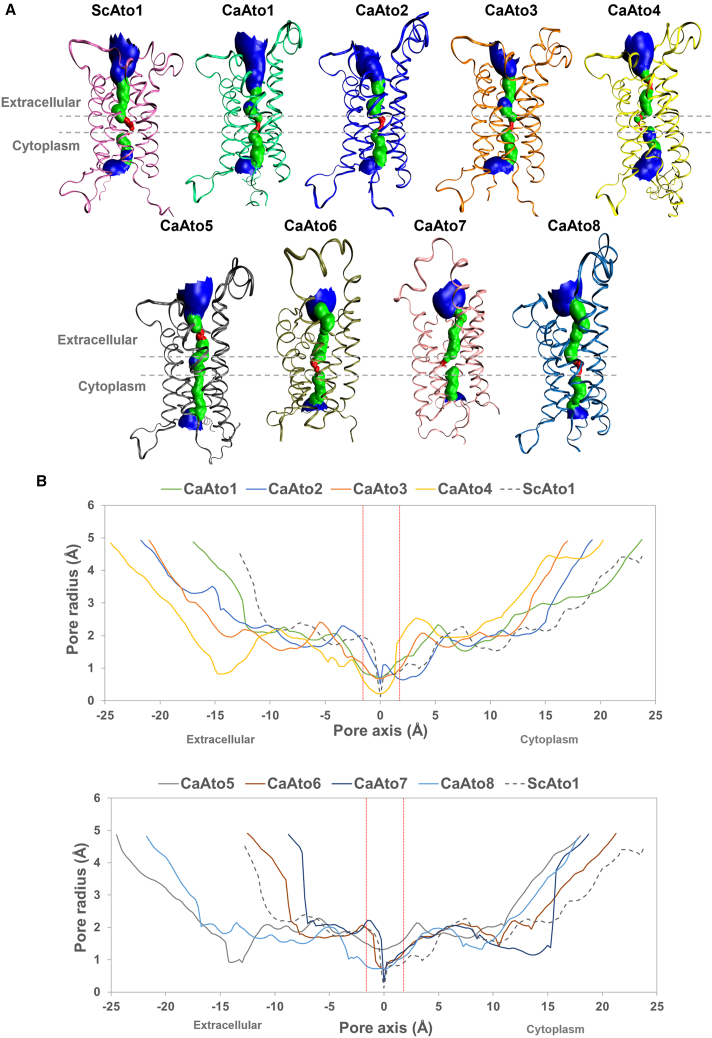
Pore 3D structure predictions and radius profile simulations along the channel axis in the Atos of *S. cerevisiae* and *C. albicans* (A) Pore 3D structure prediction of ScAto1 and CaAto1-8. The 3D structures of proteins are shown in ribbon representation, with a color scheme in which blue represents a larger pore size, green an intermediate pore size, and red a more constricted pore size as predicted by pore analysis. The horizontal dashed gray lines correspond to the constricted site where the pore radius is tight. (B) Simulations for the pore radius profiles along the channel axis in the ScAto1 and CaAto1-8. The central region of the proteins containing the constriction site is indicated by vertical red dashed lines.

The pore radii of Ato proteins in *C. albicans*, *C. glabrata*, and *C. auris* were predicted to determine the putative translocation paths for their substrates (e.g., acetate or lactate) (Figure 2). The narrowest constriction in each path is located around the FLY region (Figure 2A). Given their similarities in structure, pore size, and conserved motifs, CaAto1, CaAto2, CaAto3, and CaAto6 may transport similar substrates to ScAto1. The variations in pore radii between CaAto4, CaAto5, CaAto7, and CaAto8 compared to ScAto1 and CaAto1 (Figure 2B) suggest that these proteins might possess altered transport activities or specificities compared to ScAto1 or CaAto1.

In CaAto4, the pore seems to be blocked in this region, due to substitutions in the FLY motif. Furthermore, CaAto4 includes an additional restriction site located in the upper region of the protein, which faces the extracellular medium, specifically near the residues Phe94, Phe150, and Phe218. Also, the central pore of CaAto7 appears blocked despite conservation of the FLY motif. This blockage may be caused by variations in the amino acid residues that interact with the closest TMS. As for CaAto5, there is a widening of the pore around the FLY motif due to a substitution to TLY in this protein. In fact, the narrowest constriction site of CaAto5 is found in the upper section of the protein facing the extracellular space (Phe93, Phe149, and Phe217), similar to the second constriction site of CaAto4. The reduction of the pore radii in CaAto4 and CaAto7 might limit their uptake to only small carboxylates such as formate or might suggest signaling, rather than transport roles. Previous studies by Rendulić and colleagues (2021)^32^ found that the substitution Leu219Trp in *S. cerevisiae* Ato1 led to a reduction of the pore radius at the construction site, thereby blocking the transport of carboxylates. Therefore, a larger side chain at this position can arrest transport.

In contrast, the pore radius of CaAto5 in the FLY motif region is wider compared to ScAto1 and CaAto1, suggesting that CaAto5 may be capable of transporting larger molecules, such as succinate or citrate. Indeed, Phe98Ala and Leu219Ala mutations in ScAto1 reduce the constriction at the central FLY region and enable the transport of larger molecules, such as succinate.^32^ In addition, significant substitutions (Tyr155Phe and Leu219Val) in the FLY motif of *Cyberlindnera jadinii* Ato5, a broad-range carboxylate transporter, suggest that these residues contribute to the transport of mono-, di-, and tricarboxylates including citrate.^43^ These findings indicate that the residues within the FLY motif play a crucial role in the substrate selectivity and transport activity of Ato transporters.

We also identified significant variations between CaAto5, CaAto4, and CaAto7 in their pore radii around the NPAPLGL(M/S) and SYG(X)FW motifs (Figures 1 and 2). Also, the pore size predictions revealed distinct differences in the pore radius of CaAto8, not only at the constriction site but also in the conservation motif region (Figure 2B), which is less well conserved in CaAto8 (Figure 1). This suggests significant divergence in the transport activity of CaAto8 compared to other Ato family members.

As for the other Atos analyzed in this work, no significant variation in pore radius was observed between the Atos of *C. glabrata*, except for CgAto2 that contains two distinct constriction sites (Figures S3A–S3C). In *C. auris*, the pore radii of CauAto1-2 and CauAto3 (Figure S3D) are quite different. The extracellular loop regions that link TMSs in CauAto3 are significantly shorter than those in CauAto1-2, leading to dissimilarity in their 3D structures and pore sizes. In addition, the pore radius analyses show two constriction sites for CauAto1 (Figure S3B). An additional constriction site has been predicted for CgAto2 and CauAto1, closer to the extracellular region (Figures S3A and S3B).

### Substrate docking in Ato proteins reveals potential distinct acetate- and lactate-binding sites

**Figure 3:**
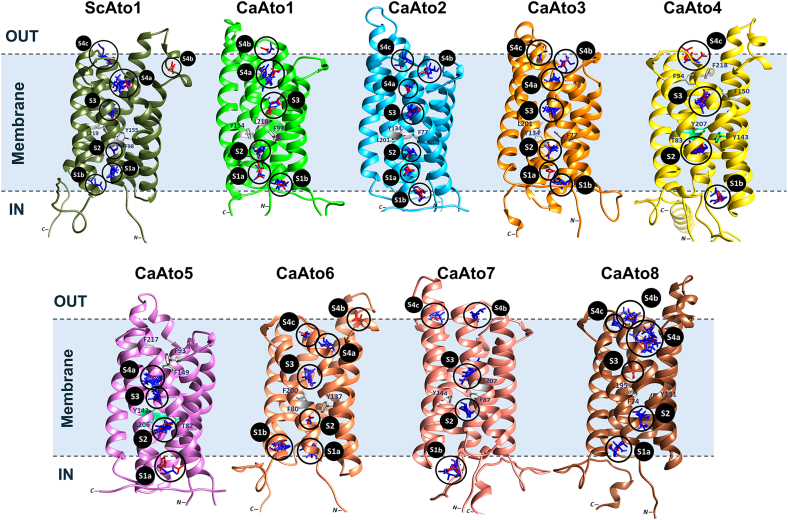
3D structure and molecular docking of ScAto1 and CaAto1-8 with acetate and lactate as substrates Predicted binding sites for acetate and lactate are indicated as S1–S4. Site S1 is located at the cytoplasmic vestibule, sites S2 and S3 are located inside the main pore, and site S4 is located at the extracellular vestibule. The positions of the N and C termini are indicated. Acetate and lactate ligands are shown in red and blue, respectively.

The crystal structures of EcSatP and CkSatP indicate a hexameric anion channel.^33^^,^^34^ Each monomer comprises six transmembrane helices and four acetate-binding sites aligned along the pore. Acetate-binding site S1 faces the cytoplasm, sites S2 and S3 are located inside the main pore surrounding the FLY motif, and site S4 faces the periplasm.^33^ The FLY motif is located centrally in the pore of the EcSatP monomer, between S2 and S3 sites, leading to the formation of an hourglass-shaped structure.^33^ We performed substrate docking of acetate and lactate for *S. cerevisiae*, *C. albicans*, *C. glabrata*, and *C. auris* Atos using AutoDock Vina from PyRx virtual screening tool. This revealed at least four predicted binding sites (S1–S4) along the pore axis of all tested proteins (Figure 3; Figure S4). The numbers and positions of predicted substrate-binding sites for CaAto1, 2, 3, and 6 were consistent with the crystal structures of the bacterial homologs EcSatP^33^ and CkSatP.^34^

CaAto4, 5, 7, and 8 contained fewer binding sites and theoretically altered binding affinities for acetate and lactate. No acetate- and lactate-binding sites were identified in the upper region of CaAto5, suggesting that this protein may not transport these substrates.

The overall estimated binding affinities of Ato proteins from *S. cerevisiae*, *C. albicans*, *C. glabrata*, and *C. auris* were largely comparable, although variations were observed at specific binding sites for acetate and lactate in certain Ato proteins (Table S3). CaAto4 and CaAto5 exhibited fewer occupied sites and showed weaker or absent interactions at specific positions, namely, S4(a, b) and S1(a) in CaAto4 and S4(b, c), and S1(b) in CaAto5. Moreover, CaAto7 and CaAto8 exhibited no acetate or lactate binding at positions S4(a), S1(a), S4(c), S1(b), and S3.

The molecular docking results for CauAto1 and CauAto3 indicated fewer and distinct acetate- and lactate-binding sites, whereas CauAto2 exhibited binding sites nearly identical to those of other Atos (Figure S4B).

### Specific CaAtos localize to the plasma membrane in the presence of mono-, di-, or tricarboxylic acids

**Figure 4:**
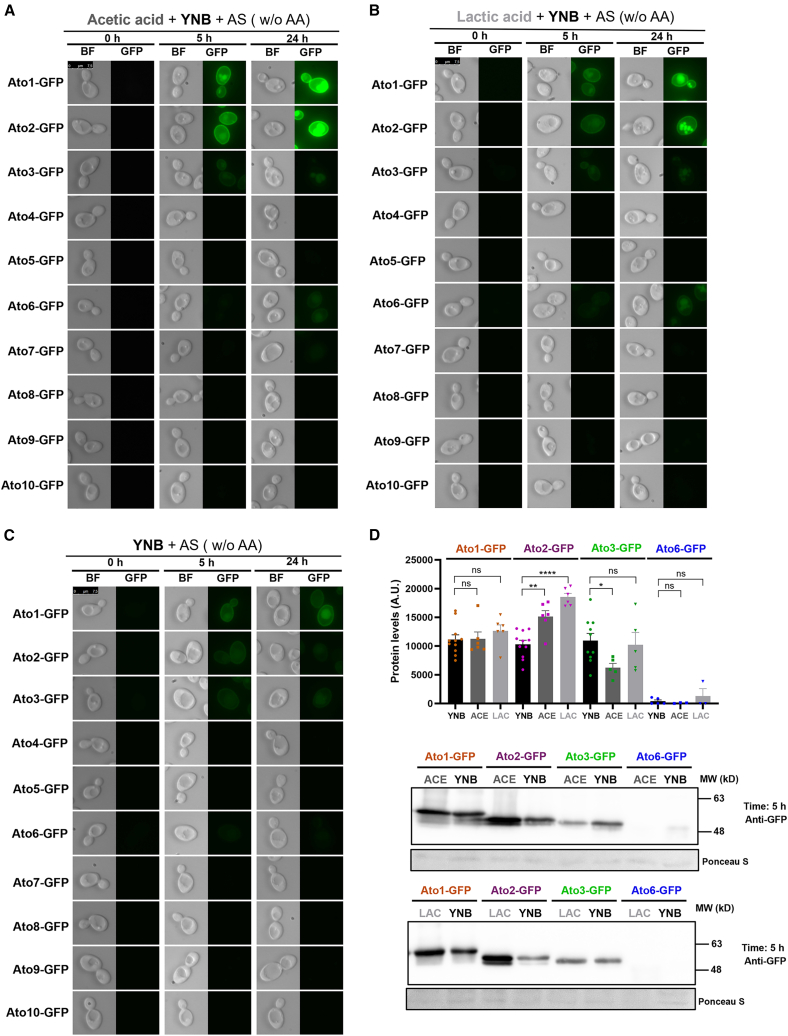
Subcellular localization and expression of Ato-GFP proteins in live *C. albicans* cells grown on different carbon sources (A–C) *C. albicans* cells were grown in 50 mL SM medium supplemented with 0.2% (w/v) glucose to exponential phase. Then they were washed with deionized water and transferred to fresh minimal media containing different carbon sources: (A) 0.1% (v/v) acetic acid pH 6.0, (B) 0.1% (v/v) lactic acid pH 5.0, and (C) without any carbon source (SM: 0.67% w/v YNB with ammonium sulfate). Samples were collected after 0-, 5- and 24-h induction, to examine cells by epifluorescence microscopy. Cell cultures were grown with aeration at 200 rpm, and 30°C. Scale bars, 7.5 μm. Data are representative of *n* = 3 independent biological replicates. BF, bright field; GFP, green fluorescent protein. (D) At the time point 5 h, protein extracts were prepared for western immunoblotting with an anti-GFP antibody. Quantification of the protein levels in arbitrary units (A.U.) was done using ImageJ software (v.1.53 k). Error bars correspond to standard error of the mean (SEM) of at least three independent experiments (Figure S8). Individual data points are plotted for all biological replicates. ns, not significant; ∗*p* = 0.0255, ∗∗*p* = 0.0014, ∗∗∗∗*p* < 0.0001 (unpaired *t* test [two-tailed] using Prism 8). Ponceau S staining was used as a transfer and loading control.

To study the expression and subcellular localization of each CaAto protein, we generated C-terminal Ato-GFP fusions for all 10 Ato proteins in *C. albicans* using CRISPR-Cas9 (Table 2). Hereafter, the resulting *C. albicans* Ato-GFP fusion proteins are referred to as Ato1-GFP to Ato10-GFP. Their expression was examined during growth in yeast nitrogen base (YNB) medium in the absence or presence of lactic or acetic acid. Only Ato1-GFP, Ato2-GFP, Ato3-GFP, and Ato6-GFP were expressed at detectable levels (Figures 4A–4C). Ato6-GFP was only detectable at the PM after prolonged growth on lactic acid for 24 h. Ato1-GFP, Ato2-GFP, and Ato3-GFP were detected at the PM after 5 h of derepression in acetic or lactic acid, as well as in the YNB control (Figures 4A–4C). After 5 h, Ato1-GFP and Ato2-GFP signals at the PM were similar, whereas Ato3-GFP was expressed at lower levels under all of the conditions tested. After 24 h, Ato1-GFP and Ato2-GFP expression increased at the PM, as did the amount of fluorescence associated with the vacuolar lumen, suggesting a rise in the turnover of these proteins. At 24 h, Ato3-GFP was localized mainly in the vacuole.

**Table 2:** List of *C. albicans* strains used in this study

Strain names	Genotype	Source
WT	SC5314	–
Ato1-GFP (FG16-19)	*ATO1-GFP/ATO1-GFP*	This study
Ato2-GFP (FG08-11)	*ATO2-GFP/ATO2-GFP*	This study
Ato3-GFP (FG12-15)	*ATO3-GFP/ATO3-GFP*	This study
Ato4-GFP (FG52-54)	*ATO4-GFP/ATO4-GFP*	This study
Ato5-GFP-Hetero (FG65-67)	*ATO5/ATO5-GFP*	This study
Ato6-GFP (FG55-56)	*ATO6-GFP/ATO6-GFP*	This study
Ato7-GFP (FG25-32)	*ATO7-GFP/ATO7-GFP*	This study
Ato8-GFP (FG59-64)	*ATO8-GFP/ATO8-GFP*	This study
Ato9-GFP (FG71-73)	*ATO9-GFP/ATO9-GFP*	This study
Ato10-GFP (FG74-76)	*ATO10-GFP/ATO10-GFP*	This study
Ato1-GFP *ato2Δ/Δ* (RA42-44)	*ATO1-GFP/ATO1-GFP ato2Δ::NAT/ato2Δ::NAT*	This study
Ato3-GFP *ato1Δ/Δ* (RA45-47)	*ATO3-GFP/ATO3-GFP ato1Δ::NAT/ato1Δ::NAT*	This study
Ato3-GFP *ato2Δ/Δ* (RA48-50)	*ATO3-GFP/ATO3-GFP ato2Δ::NAT/ato2Δ::NAT*	This study
Ato2-GFP *ato1Δ/Δ* (RA52-54)	*ATO2-GFP/ATO2-GFP ato1Δ::NAT/ato1Δ::NAT*	This study
Ato1-GFP *ato3Δ/Δ* (PA19-23)	*ATO1-GFP/ATO1-GFP ato3Δ::NAT/ato3Δ::NAT*	This study
Ato2-GFP *ato3Δ/Δ* (PA24-26)	*ATO2-GFP/ATO2-GFP ato3Δ::NAT/ato3Δ::NAT*	This study
*ato1Δ/Δ* (RA55-61)	*ato1Δ/Δ*	Alves et al.^41^
*ato1-10Δ/Δ(*RA239-241)	*ato1Δ/Δ ato2Δ/Δ ato3Δ/Δ ato4Δ/Δ ato5Δ/Δ ato6Δ/Δ ato7Δ/Δ ato8Δ/Δ ato9Δ/Δ ato10Δ/Δ*	Alves et al.^41^

To further investigate the expression and localization patterns of Ato-GFP fusion proteins, the full collection was also analysed in synthetic complete medium supplemented with a broad range of mono-, di-, or tricarboxylic acids including formic acid (0.1% v/v, pH 5.0), butyric acid (0.1% v/v, pH 5.0), succinic acid (0.1% w/v, pH 5.0), pyruvic acid (0.1% w/v, pH 6.0), malic acid (0.1% w/v, pH 6.0), and citric acid (0.1% w/v, pH 6.0) (Figure S10A–S10G). Ato1-GFP, Ato2-GFP, Ato3-GFP, and Ato6-GFP were expressed at detectable levels in the presence of formic and succinic acid. Ato1-GFP and Ato2-GFP were localized at the PM after 16 h of incubation in either formic or succinic acid. After 40 h, Ato1-GFP and Ato2-GFP levels increased at the PM, as did the amount of fluorescence detected in the vacuolar lumen. In contrast, Ato3-GFP and Ato6-GFP were expressed at lower levels under the same conditions (Figures S10A–S10C).

In the presence of butyric acid, detectable expression of Ato1-GFP, Ato2-GFP, Ato3-GFP, and Ato6-GFP was notably delayed. Ato1-GFP and Ato2-GFP localized to the PM only after 20 h incubation in butyric acid, with a slight increase in signal intensity by 40 h. Ato3-GFP and Ato6-GFP were also expressed at lower levels under this condition (Figure S10B).

Pyruvic acid induced detectable expression of several Ato-GFP fusion proteins. Ato1-GFP and Ato2-GFP were highly expressed after 16 h of incubation and remained localized at the PM after 40 h. Under these conditions, Ato6-GFP displayed higher signal intensity than observed with the other tested carboxylic acids tested. In addition, Ato3-GFP and Ato7-GFP were expressed at low levels in the presence of pyruvic acid (Figure S10D).

In the presence of citric acid, few members of the Ato-GFP protein family exhibited detectable expression. Ato1-GFP showed low levels of expression at 18 and 20 h, with an increase observed only between 24 and 40 h. In contrast, Ato2-GFP displayed higher expression at 18 h, which increased and persisted at the PM over time. Ato6-GFP consistently showed a weak GFP signal at the PM during the incubation. Moreover, Ato3-GFP and Ato7-GFP were undetectable at the PM; only faint vacuolar fluorescence appeared after 24 h in the presence of citric acid (Figure S10F).

In the presence of malic acid, Ato1-GFP expression was detected earlier than the other Ato-GFP fusion proteins, with signal observed at 16 h. By 18 h, Ato1-GFP, Ato2-GFP, and Ato3-GFP were localized to the PM. Ato1-GFP and Ato2-GFP expression levels increased over time and persisted up to 40 h, whereas Ato3-GFP exhibited the same pattern but with lower signal intensity (Figure S10E).

Other Ato-GFP fusion proteins, including Ato4-GFP, Ato5-GFP, Ato8-GFP, Ato9-GFP, and Ato10-GFP, exhibited no detectable expression and no detectable GFP signal under the carboxylate conditions tested. As a control, the entire collection of Ato-GFP fusions were also analysed in synthetic minimal (SM) medium supplemented with 0.2% glucose. No detectable GFP signal was observed in any of the strains under this condition (Figure S10G).

To further assess the effects of lactic and acetic acid on the expression levels of Ato1-GFP, Ato2-GFP, Ato3-GFP, and Ato6-GFP, western blot analyses were performed using total protein extracts (Figure 4D). Quantification of protein levels revealed that neither acetic nor lactic acid significantly affected the amount of Ato1-GFP, relative to the YNB control. This was rather surprising given that we have directly demonstrated that CaAto1 is the main transporter responsible for radiolabeled acetate uptake.^41^ On the other hand, carboxylic acids induced a significant increase in the expression of CaAto2 (Figure 4D), suggesting that besides CaAto1, CaAto2 might play a role in the uptake or utilization of weak organic acids. Acetic acid also led to a decrease in Ato3-GFP abundance, but lactic acid did not change Ato3-GFP expression levels compared to the YNB control condition (Figure 4D). Ato6-GFP showed low levels of expression at 5 h with no considerable differences between the tested conditions.

Overall, only CaAto1, CaAto2, CaAto3, and CaAto6 were detected by both fluorescence microscopy and western blotting, supporting our *in silico* predictions that these proteins are the major CaAto family members involved in carboxylate transport. As the remaining CaAto transporters are not expressed under carboxylate-related growth conditions and show reduced or altered acetate- or lactate-docking sites, they may instead be involved in the uptake of substrates other than the carboxylic acids tested.

### Deletion of *CaATO1* affects the localization and expression of Ato2-GFP and Ato3-GFP

**Figure 5:**
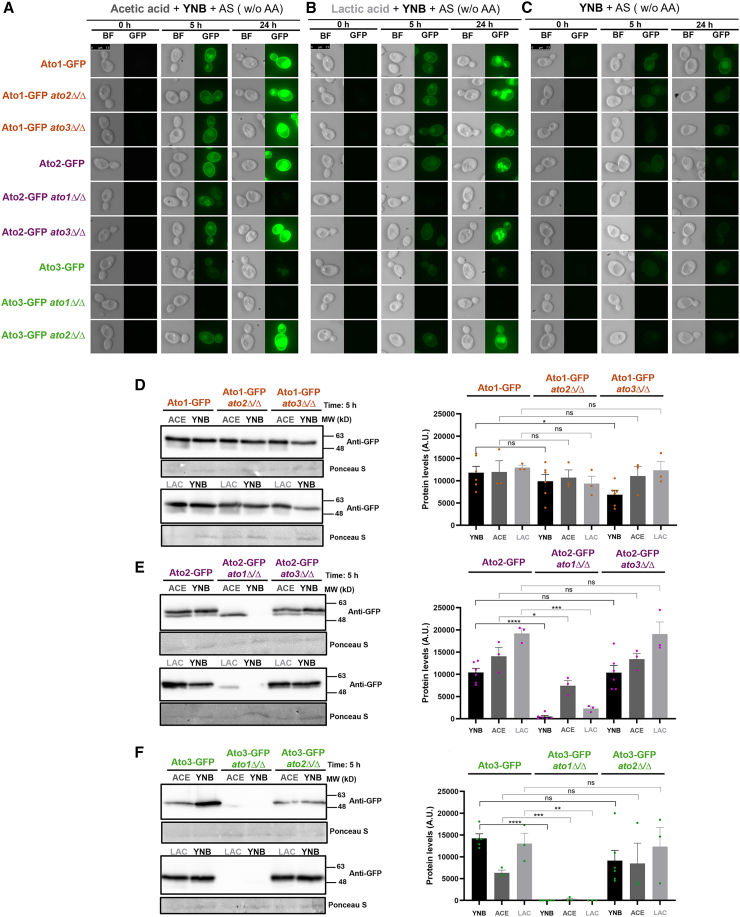
Subcellular localization and expression of the indicated Ato-GFP fusion proteins in WT and *ato*-mutant strains of *C. albicans* (A–C) *C. albicans* cells were grown in 50 mL SM medium supplemented with 0.2% (w/v) glucose to exponential phase. Then they were washed then with deionized water and transferred to fresh minimal media containing different carbon sources: (A) 0.1% (v/v) acetic acid pH 6.0, (B) 0.1% (v/v) lactic acid pH 5.0, and (C) without any carbon source (SM: 0.67% w/v YNB with ammonium sulfate). Samples were collected after 0-, 5- and 24-h induction, to examine cells by epifluorescence microscopy. Cell cultures were grown with aeration at 200 rpm, and 30°C. Note: images for the WT strains (Ato1-3-GFP; A–C, rows 1, 4, and 7) are reproduced from Figures 4A–4C to allow for a direct side-by-side comparison with the mutants from the same experiment. Scale bars, 7.5 μm. Data are representative of *n* = 3 independent biological replicates. BF, bright field; GFP, green fluorescent protein. (D–F) At the time point 5 h, protein extracts were prepared for western immunoblotting with an anti-GFP antibody. Quantification of the protein levels in arbitrary units (A.U.) was done using ImageJ software (v.1.53 k). Error bars correspond to standard error of the mean (SEM) of at least three independent experiments (Figure S8). Individual data points are plotted for all biological replicates. ns, not significant; ∗*p* = 0.0183–0.0455, ∗∗*p* = 0.0014–0.0054, ∗∗∗*p* = 0.0001–0.0008, ∗∗∗∗*p* < 0.0001 (unpaired *t* test [two-tailed] using Prism 8). Ponceau S staining was used as a transfer and loading control.

To explore potential cooperative interactions between CaAto proteins, we constructed *ato1*, *ato2*, or *ato3* deletions in the Ato1-GFP, Ato2-GFP, and Ato3-GFP backgrounds in *C. albicans* (Table 2). The expression and subcellar localization of Ato-GFP proteins in these strains were examined in response to the presence of acetic or lactic acid (Figure 5). The deletion of *CaATO2* did not interfere with Ato1-GFP or Ato3-GFP expression under any of the conditions tested as demonstrated by GFP fluorescence (Figures 5A–5C) and western blotting (Figure 5D). However, deleting *CaATO3* slightly affected the expression of Ato1-GFP in YNB (Figure 5D), but did not influence Ato2-GFP protein levels under the conditions tested (Figure 5F).

Most interestingly, under all conditions tested, the deletion of *CaATO1* had a pronounced impact on the expression and PM localization of Ato2-GFP and Ato3-GFP (Figures 5A–5C, rows 5 and 8). The *ato1Δ/Δ* mutant exhibited Ato2-GFP localization at the cortical and nuclear endoplasmic reticulum (ER) after 5 h, of transcriptional induction, followed by complete abolishment of Ato2-GFP expression after 24 h in the presence of acetic acid. Co-staining with DAPI revealed that the GFP signal in the Ato2-GFP *ato1Δ/Δ* strain exhibited a ring-like pattern surrounding the DAPI-stained nucleus, consistent with localization to ER associated with the nuclear envelope (Figure S11). Moreover, the deletion of *CaATO1* resulted in a complete loss of Ato2-GFP and Ato3-GFP expression in YNB and in response to lactic acid (Figures 5B and 5C, lines 5 and 8). These results were consistent with the western blot analysis, which showed either no detectable bands or only faint bands for both Ato2-GFP and Ato3-GFP, under these conditions. Also, acetic acid led to a decrease in Ato3-GFP expression levels, whereas lactic acid led to a slight increase in Ato6-GFP abundance, but these changes were statistically non-significant.

In addition, western blotting revealed notable effects also upon Ato2-GFP expression in the presence of acetic and lactic acid (Figure 5E). Ato2-GFP was detected as two bands, possibly representing different post-translationally modified forms of Ato2-GFP. However, in the Ato2-GFP *ato1 Δ/Δ* strain, only the lower band was observed in response to acetic or lactic acid (Figure 5E). This suggests that CaAto1 might regulate CaAto2 by controlling (directly or indirectly) its post-translation modification, although the specific nature of this modification and underlying mechanism remain to be elucidated. *In silico* analyses predict that CaAto2 contains 20 potential phosphorylation sites including 12 serine and 8 threonine residues (Figure S9) and no predicted glycosylation sites. Four serine residues at positions 6, 9, 13, and 28 showed approximately maximum scores (1.000), suggesting a high probability of phosphorylation. Importantly, these residues are localized within the N-terminal region of the protein. Several phosphorylation sites have been reported in the N-terminal region of Ato proteins in *S. cerevisiae*.^44^ These observations suggest that CaAto2 might undergo post-translational modification, potentially via phosphorylation. Noticeably, after 5 h in acetic acid, the presence of the lower Ato2-GFP band correlated with fluorescence in the ER (Figure 5A, line 5; Figure S11), suggesting that unmodified Ato2-GFP fails to be targeted to the PM and that this modification probably occurs at the ER. In the absence of CaAto1, neither form of Ato2-GFP was observed under conditions lacking acetic or lactic acid (Figure 5E, YNB control), reflecting the lack of fluorescence under these conditions at 24 h (Figures 5B and 5C, line 5). The same was true for Ato3-GFP in *ato1 Δ/Δ* cells (Figures 5B and 5C, line 8; Figure 5F). However, the Ato2-GFP banding pattern was not altered by treatment with calf intestinal alkaline phosphatase (not shown), suggesting that alternative post-translational modifications may underlie the partial ER retention of Ato2-GFP.

Taken together, the data suggest that CaAto1 is required for the stable expression and proper localization of CaAto2 and CaAto3 in *C. albicans*. This would be compatible with physical interactions among CaAto1, CaAto2, and CaAto3, potentially leading to the formation of heterohexameric complexes in *C. albicans.* This possibility is discussed further later.

### CaAto1 primarily transports acetate

We previously demonstrated that CaAto1 is the major acetate transporter in *C. albicans*^41^: *ato1Δ/Δ* cells display a dramatic reduction in acetate uptake and a significant growth defect on acetate, compared to wild-type (WT) controls.^41^ Here, we further characterized CaAto1 by exploring its substrate specificity and determining its affinity for carboxylates beyond acetate via substrate competition assays. *C. albicans* cells expressing Ato1-GFP (Ato1-GFP reference strain, Table 2) were grown to exponential phase in SM medium (with 0.2% glucose) and subsequently derepressed for 5 h in SM medium containing acetate (0.1% v/v, pH 6.0). The uptake of radiolabeled acetate (0.1 mM) was then measured in the presence of a 100-fold excess of individual non-labeled carboxylates (10.0 mM): lactate, pyruvate, formate, butyrate, succinate, malate, citrate, and acetate as a control.

**Figure 6:**
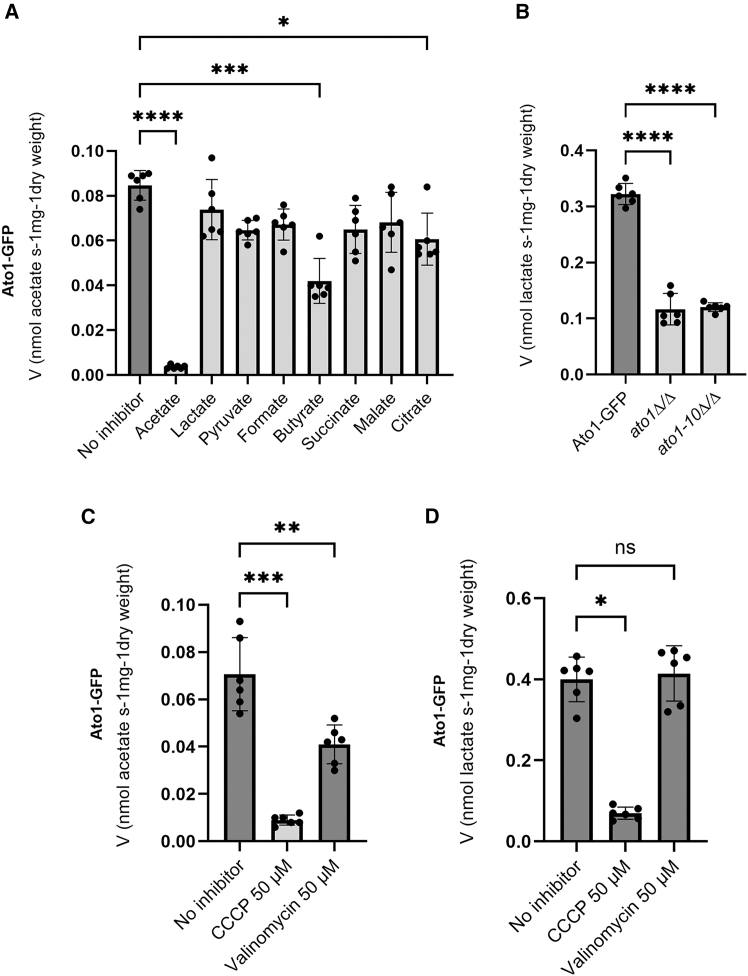
Acetate or lactate uptake and transport energetics of *C. albicans* Ato1-GFP, *ato1Δ/Δ*, and *ato1-10Δ/Δ* strains *C. albicans* cells following 5-h derepression in SM medium containing 0.1% (v/v) acetate (pH 6.0). (A) Transport specificity of radiolabeled [^14^C]acetate (0.1 mM, pH 6.0) in Ato1-GFP in the presence of unlabeled acetate, lactate, pyruvate, formate, butyrate, succinate, malate, and citrate (10 mM). (B) Uptake of [^14^C]lactate (2.0 mM, pH 6.0) in Ato1-GFP, *ato1Δ/Δ*, and *ato1-10Δ/Δ* strains. (C and D) Transport energetics: effect of CCCP and valinomycin on the uptake of 0.1 mM [^14^C]acetate and 2 mM [^14^C]lactate in Ato1-GFP. Data represent standard deviation (SD) from at least three independent experiments (*n* = 6). Individual data points are plotted for all biological replicates. Data were tested for normality prior to analysis. Statistical analysis: Kruskal-Wallis test (A and D); Welch’s one-way ANOVA with Dunnett’s T3 post hoc test (B and C). ns, not significant; ∗*p* = 0.0189–0.0307, ∗∗*p* = 0.0063, ∗∗∗*p* = 0.0003–0.0004, ∗∗∗∗*p* < 0.0001.

As expected for CaAto1,^41^ the addition of excess non-labeled acetate completely inhibited the uptake of the radiolabeled acetate (Figure 6A). Excess non-labeled butyrate reduced radiolabeled acetate uptake by approximately 50% whereas the other carboxylates tested produced only modest inhibition, ranging from 15% to 20%. These results indicate that CaAto1 is relatively specific for high-affinity acetate transport, with substantially lower affinity (*K*_i_ > 10 mM) for most other carboxylates tested. To define the energetic requirements of CaAto1-mediated acetate uptake, we also evaluated uptake in the Ato1-GFP strain in the presence of specific protonophores or ionophores. Carbonyl cyanide *m*-chlorophenylhydrazone (CCCP) was employed to collapse the total proton motive force and valinomycin to dissipate the membrane electrical potential (Δψ) (Figure 6C). While CCCP nearly fully inhibited CaAto1-mediated acetate uptake, valinomycin reduced it only by approximately 35%. This strongly suggests that the CaAto1 transporter is driven specifically by the proton motive force component of the electrochemical gradient, specifically relying on a high pH gradient rather than the membrane potential.

We also investigated CaAto1 transport activity by measuring lactate transport kinetic parameters in Ato1-GFP, *ato1Δ/Δ*, and *ato1-10Δ/Δ* strains (Table 2). This revealed that, in Ato1-GFP cells, lactate uptake velocity increased linearly in a non-saturable fashion over the range of concentrations used. In contrast, practically no lactate uptake was recorded in *ato1Δ/Δ* or *ato1-10Δ/Δ* strains (Figure 6B; Figure S12A). Interestingly, this Ato1-dependent lactate uptake was also inhibited by CCCP (Figure 6C), suggesting that even this low-affinity, minor transport activity of Ato1 seems to take place via H^+^ symport.

Notice that prior to the above-described experiments we confirmed that GFP tagging did not affect CaAto1 function by comparing the uptake of radiolabeled acetate (4 mM) in WT and Ato1-GFP strains. No significant difference in uptake was observed between the two strains (Figure S12B), indicating that the GFP tag does not influence the ability of CaAto1 to transport acetate.

### Vertebrate ATOs are characterized by transport domain duplications and a gene fusion event

**Figure 7:**
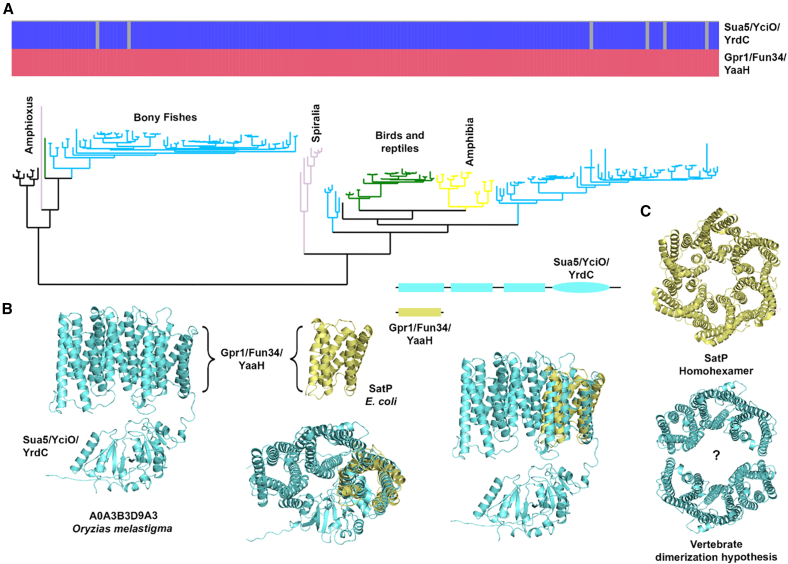
Vertebrate Ato phylogeny and structure (A) Phylogeny and domain composition of vertebrate Ato proteins. Tree branches are colored based on taxonomy. Full metazoan phylogeny can be seen in Figure S6. (B) AlphaFold 2.0 structure prediction of a vertebrate Ato protein (cyan) and crystal structure of SatP (yellow). The triplication of the Gpr1/Fun34/YaaH domain is apparent when structures are superimposed and schematically presented in the cartoon (rectangles, Gpr1/Fun34/YaaH; eclipse, Sua5/YciO/YrdC). (C) Dimerization hypothesis of the vertebrate Ato homologs based on the experimentally determined SatP hexamer.

Beyond functional analyses in *C. albicans*, we investigated the evolutionary conservation of the Ato/AceTr family. Previous taxonomic distribution studies have suggested that AceTr/Ato proteins are present in prokaryotic and eukaryotic microbes,^26^^,^^43^ but not in Metazoa. Those studies employed BLASTp searches, using known Ato proteins as baits (SatP from *E. coli,* AceP from *Methanosarcina acetivorans,* Ato1/Ady2 from *S. cerevisiae,* or Atos from *C. jadinii*)*.* Here*,* we used a sensitive hmmsearch together with the PFAM HMM-profile (PF01184) of the AceTr transporter family to collect fungal and metazoan sequences. This analysis revealed that AceTr/Ato family members are widespread within Bilateria and particularly in vertebrates (Figure 7A; Figures S5 and S7). Vertebrate ATOs form two clades with representatives from every major lineage, except mammals (Figure 7). A small number of protostome sequences can be identified within the vertebrate clade, suggesting possible horizontal gene transfer. Notably, vertebrate ATOs and their close invertebrate homologs are *circa* 900 amino acids in length, contrasting with the 200–300 amino acid length of their fungal and prokaryotic counterparts (Figure 1).^33^^,^^34^ Surprisingly, almost all of the vertebrate sequences that we identified have a C-terminal fusion to a Sua5/YciO/YrdC domain in addition to the Gpr1/Fun34/YaaH domains that characterize the AceTr/Ato transporter family (Figure 7; Figure S6). This domain is not present in the bacterial SatP (Figure 6) or in the protostome and fungal clades (Figure S7). Sua5/YciO/YrdC proteins are annotated as threonyl-carbamoyl-AMP synthetases, which attach a threonyl-carbamoyl group to A37 (t6A37) of a tRNA.^45^ This tRNA modification is common for tRNAs that have A/U-rich anticodons, helping to stabilize their codon-anticodon interactions.^46^ Loss of threonyl-carbamoyl-AMP synthetase (Sua5) leads to increased rates of translational frameshifting and stop codon readthrough in yeast, underlining the importance of the t6A37 modification for translational fidelity.^47^

Comparing the experimentally resolved SatP structure from *E. coli*^33^ with the AlphaFold 2.0^48^ predictions of some vertebrate Ato proteins revealed that they consist of three Gpr1/Fun34/YaaH domains (six helices each) fused in tandem (Figure 7B). HMM-scan for the Gpr1/Fun34/YaaH HMM-profile in vertebrate sequences returns three domain hits for some of them (Figure S6). Structural investigation of proteins that produce less than three hits revealed that they possess three copies of the six-helix Gpr1/Fun34/YaaH fold (Figure S6). TM-align superposition of the vertebrate A0A3B3D9A3 from *Oryzias melastigma* (chordate fish) with SatP revealed strong structural similarity with an root-mean-square deviation of 2.53 Å and a TM-score of 0.82. Superimposed structures of the two proteins are presented in Figure 6. Interestingly, SatP crystals have revealed a homohexameric structure^33^ (Figure 7C). Observing the duplicated Gpr1/Fun34/YaaH domains of vertebrate Atos, we hypothesize that two interacting protomers could be arranged in a six-domain ring similar to the one formed by SatP. Hence, cross-kingdom conservation of the six-domain ring structure of Ato proteins suggests that its functional relevance persists in vertebrate homologs.

## Discussion

This study focused on the identification of carboxylate transporters belonging to the AceTr family in *C. albicans.* The expansion of the *ATO* gene family (*ATO1–ATO10*) suggests a specialized system for organic acid uptake, likely reflecting an evolutionary adaptation to utilize alternative carbon sources in diverse host niches. Indeed, our results show that the Ato protein family in other pathogenic *Candida* species is also expanded and functionally diverse, albeit to a lower extent. We found that key motifs including NPAPLGL and FLY are conserved among *Candida* Ato homologs consistent with previous reports that AceTr proteins share the NPAPLGL(M/S) motif at the start of the first TMS.^25^^,^^26^^,^^49^ However, we further demonstrate that significant variations exist in pore constriction and acetate/lactate docking, indicating that individual Ato proteins likely differ in substrate affinity and specificity. In particular CaAto1, CaAto2, CaAto3, and CaAto6 appear to be the principal Atos involved in monocarboxylate transport.

Expression analyses indicated that CaAto1, CaAto2, CaAto3, and CaAto6 display distinct PM localization and expression levels, in the presence of various carboxylate substrates, in good line with the *in silico* predictions. CaAto1 and CaAto2 are consistently highly expressed and localized to the PM in the presence of all tested mono-, di-, and tricarboxylic acids. In contrast, CaAto3 and CaAto6 exhibit consistently low expression and minimal PM localization under the same conditions. Other CaAto proteins, including Ato4, Ato5, Ato7, Ato8, Ato9, and Ato10, were not detected under our experimental conditions and thus may not be involved in the uptake of the tested carboxylates, suggesting that they rather transport different substrates or act under specific environmental conditions. In addition to Ato1, which was shown here to be the primary transporter responsible for acetate uptake in *C. albicans*,^41^ Ato2 may also play an important role in the uptake of acetate or other non-fermentable carbon sources, as suggested by its consistent localization at the PM during growth on acetic or lactic acid.

Interestingly, the localization of CaAto2 and CaAto3 at the PM was dependent on CaAto1, which is consistent with the idea that functional Ato proteins may assemble as hetero-oligomeric complexes in *C. albicans*. This is in line with the observation that in *S. cerevisiae* ScAto1 and ScAto2 interact physically, while there are no interactions between ScAto1 and ScAto3, ScAto2, and ScAto3. In fact, ScAto1 and ScAto3, but not ScAto2, are also capable of forming homodimers.^50^^,^^51^ The finding that the bacterial homolog SatP functions as a homohexamer^33^ further suggests that homo- or hetero-oligomerization may be a conserved feature required for the proper activity and regulation of Ato-like transporter proteins. In case Ato proteins in *C. albicans* form heterohexameric complexes, Ato1 seems to be a central and essential subunit providing a stable core within the complex, while the remaining Ato proteins may be substituted in a condition-dependent manner to facilitate rapid adaptation and maybe pathogenicity of *C. albicans*.

We have provided formal evidence that CaAto1 mediates acetate uptake via H^+^ symport with a restricted substrate-recognition profile. This finding is consistent with other members of the AceTr family, including *S. cerevisiae* Ato1/Ady2, *E. coli* SatP, and *M. acetivorans* AceP, which have been characterized as proton-dependent monocarboxylate permeases.^30^^,^^31^^,^^52^ Interestingly, the low-affinity transport of lactate by CaAto1 occurs through the same H^+^-symport mechanism as acetate, indicating that CaAto1 employs H^+^-coupled mechanism regardless of the substrate affinity. Notably, in *C. albicans*, lactate uptake is predominantly mediated by the Jen1 transporter, identified through its homology to *S. cerevisiae* Jen1^53^ and characterized as the primary H^+^-symporter responsible for uptake of lactate, pyruvate, and propionate.^54^ Therefore, CaAto1 might only additionally contribute to lactate accumulation as a minor transporter. Overall, our findings suggest that CaAto1 acts as a proton symporter with high-affinity for acetate uptake, while retaining a minor, proton-dependent capacity to transport other carboxylates, potentially contributing to the metabolic flexibility of *C. albicans* in fluctuating host environments.

The observation that members of the Ato family are found in vertebrates, and that some exist as fusions with the Sua5 enzyme, points to a potential evolutionary conservation of these proteins. Although the mechanistic implications of this fusion event remain unclear, the cytoplasmic region of vertebrate Ato members might catalyze a reaction similar to Sua-mediated carbamoyl-AMP synthesis. Therefore, its exact biochemical function in vertebrates needs further investigation. This fusion may represent a mechanism that could couple substrate import to metabolic assimilation, thereby potentially increasing the efficiency of downstream reactions.

In conclusion, our findings suggest that Ato proteins in *C. albicans* combine functional specialization with conserved structural features. These proteins probably form complexes, and distinct members of the family may transport specific substrates under defined circumstances. This work advances understanding of the structure and potential function of Ato proteins in *C. albicans* and provides a foundation for future studies in higher eukaryotes.

### Limitations of the study

While this study offers significant insights into CaAto protein architecture, conservation, and function, further work will be required to fully elaborate the roles of each member of this complex transporter family in *C. albicans* during commensalism and infection. This is complicated by the likelihood of functional complementation, and the possibility that Ato proteins assemble into hetero-oligomeric complexes. These issues lie beyond the scope of the present study but highlight directions for future work.

## Resource availability

### Lead contact

Requests for further information and resources should be directed to and will be fulfilled by the lead contact, Sandra Paiva (spaiva@bio.uminho.pt).

### Materials availability

All reagents generated in this study, including *C. albicans* strains, are available from the lead contact upon request.

### Data and code availability

• The raw western blot and microscopy data associated with this study have been deposited and are publicly available as of the date of publication at RepositórioUM: https://doi.org/10.34622/datarepositorium/EQT3Q4.
• This paper does not report original code.
• Any additional information required to reanalyze the data reported in this paper is available from the lead contact upon request.

## Acknowledgments

This work was supported by the MetaFungal project PTDC/BIA-MIC/5246/2020 (MIC/5246/2020 http://doi.org/10.54499/PTDC/BIA-MIC/5246/2020), through the “Fundação para a Ciência e a Tecnologia” (10.13039/501100001871FCT), and by project TransFactory (COMPETE2030-FEDER-00750700) co-financed by ERDF through COMPETE 2030. Work carried out at the University of Minho (Centre of Molecular and Environmental Biology-CBMA) was supported by the Contrato-Programa UID/04050/2025, funded by national funds through the FCT. Work at KU Leuven (Laboratory of Molecular Cell Biology) was supported by a grant from the Fund of Scientific Research Flanders (10.13039/501100003130FWO # G0C0622N) and by a fund from the KU Leuven research fund (C1 project #C14/22/075 to P.V.D.). F.G. acknowledges 10.13039/501100001871FCT for the 2023.03135.BD PhD grant (https://doi.org/10.54499/2023.03135.BD) and Erasmus+ Program for supporting her stay at KU Leuven (Belgium). P.A. acknowledges 10.13039/501100001871FCT for 2024. 03178.BDANA PhD grant. Y.P. was supported by a Fondation Santé Sidney Altman Scholarship. J.A. acknowledges 10.13039/501100001871FCT for the PD/BD/150584/2020 (https://doi.org/10.54499/PD/BD/150584/2020). A.G.-G. acknowledges 10.13039/501100001871FCT for 2021. 08564.BD PhD grant (https://doi.org/10.54499/2021.08564.BD). W.V.G. was supported by the KU Leuven Research Council grant (C14/22/075). J.N. was supported by a fellowship from the Fund of Scientific Research Flanders (1S18121N). A.J.P.B. was supported by a program grant from the 10.13039/501100000265UK Medical Research Council (MR/M026663/2), a Welcome Investigator Award (224323/Z/21/Z), the 10.13039/501100019690Medical Research Council Centre for Medical Mycology at the University of Exeter (MR/N006364/2 and MR/V033417/1), and the 10.13039/501100000272National Institute for Health and Care Research (NIHR) Exeter Biomedical Research Center. The authors thank Inês Ribeiro (CBMA) for expert technical assistance with DAPI staining. Elements of the graphical abstract were created using BioRender.com.

## Author contributions

F.G., data curation, formal analysis, investigation, methodology, software, visualization, writing – original draft, and writing – review and editing; P.A., formal analysis, investigation, methodology, and visualization; C.B.-A., formal analysis, investigation, methodology, visualization , and writing – review & editing; Y.P., data curation, formal analysis, software, visualization, and writing – review and editing; J.A., investigation and review and editing; V.F., investigation; R.A., investigation; A.G.-G., investigation; M.C., formal analysis, validation, and writing – review and editing; W.V.G., investigation and methodology; J.N., investigation and methodology; A.J.P.B., conceptualization, formal analysis, validation, and writing – review and editing; P.V.D., conceptualization, funding acquisition, methodology, resources, supervision, and writing – review and editing; A.A.P., conceptualization, formal analysis, validation, and writing – review and editing; G.D., conceptualization, formal analysis, validation, writing – review and editing, and methodology; I.S.-S., conceptualization, formal analysis, investigation, methodology, funding acquisition, supervision, validation, and writing – review and editing; S.P., conceptualization, formal analysis, funding acquisition, resources, supervision, validation, writing – review and editing, methodology, and project administration.

## Declaration of interests

The authors declare no competing interests.

## STAR★Methods

### Key resources table

**Table:** 

REAGENT or RESOURCE	SOURCE	IDENTIFIER
**Antibodies**		

Anti-GFP (monoclonal, mouse IgG1κ, clones 7.1 and 13.1)	Roche	Cat# 11814460001**;** RRID: AB_390913
Anti-mouse-IgG -peroxidase produced in rabbit	Sigma	Cat# A9044; RRID: AB_258431

**Chemicals, peptides, and recombinant proteins**		

Yeast extract	Panreac Applichem	403687
Bacto Peptone	Thermo Fisher	211677
D (+)-glucose anhydrous	scharlau	GL01271000
D- (+)-maltose monohydrate	Fisher scientific	BP684-500
bacteriological agar	GRISP	GCM25.0500
ammonium sulfate (A.S)	Panreac Applichem	131140.1211
yeast nitrogen base without amino acids and ammonium Sulfate (YNB)	Fisher Scientific	233520
Synthetic Complete Mixture (Kaiser) Drop-Out: -HIS -URA	Formedium	DSCK1015
acetic acid	Sigma-Aldrich	A6283
lactic acid	Sigma-Aldrich	27715
succinic acid	Sigma-Aldrich	S7501
butyric acid	Sigma-Aldrich	B103500
citric acid	Fisher Scientific	1072370
sodium pyruvate	Panreac Applichem	A1530
malic acid	Sigma-Aldrich	240176
formic acid	Merck	1.00264
sodium chloride (NaCl)	Honeywell/Fluka	71380
sodium hydroxide (NaOH)	Merck	1064981000
disodium hydrogen phosphate (Na_2_HPO_4_)	Scharlau	SO03371000
potassium dihydrogen phosphate (KH_2_PO_4_)	Scharlau	PO02601000
potassium chloride (KCl)	PanReac AppliChem	131494.1211
trichloroacetic acid (TCA)	Sigma-Aldrich	T6399
tris(hydroxymethyl)aminomethane (Trizma base)	Sigma-Aldrich	RDD008
ethylenediaminetetraacetic acid (EDTA)	Honeywell/Fluka	03610
sodium dodecyl sulfate (SDS)	Sigma-Aldrich	436143
glycerol	Sigma-Aldrich	G7757-1 L
bromophenol blue	Sigma-Aldrich	B0126-25G
2-mercaptoethanol	Sigma-Aldrich	M3148-25ML
Ponceau S	Sigma-Aldrich	81460-5G
WesternBright ECL HRP substrate kit	Advansta	K-12045-D50
bis-acrylamide (37.5:1)	Fischer Scientific	BP14101-1 L
[1-^14^C]-acetate	Perkin Elmer	NEC084A001MC
L-[^14^C-(U)]-lactate	NEN Radiochemicals	PELSNEC599050UC
nourseothricin (clonNAT)	Jena Bioscience	AB-101 L
ampicillin	Formedium	AMP100
CloneAmp HiFi PCR Premix	Takara Bio	639298
Q5 High-Fidelity DNA Polymerase	New England Biolabs	M0491S
*MssI* (PmeI) restriction enzyme	Thermo Fisher	FD1344
scintillation fluid (Opti-phase HiSafe II)	PerkinElmer	1200-436
carbonyl cyanide *m*-chlorophenylhydrazone (CCCP)	Sigma-Aldrich	C2759
valinomycin	Fluka	94675
paraformaldehyde (PFA)	Merck	30525-89-4
DAPI (4′,6-diamidino-2-phenylindole, dilactate)	Thermo Fisher	D3571
L-Histidine	Formedium	DOC0144
L-Uracil	Formedium	DOC0213

**Critical commercial assays**		

NZYGelpure kit for the purification of DNA	NZYGelpure	MB01102

**Deposited data**		

Raw data of Western Blots	This study	RepositóriUM: https://doi.org/10.34622/datarepositorium/EQT3Q4
Raw microscopy images	This study	RepositóriUM: https://doi.org/10.34622/datarepositorium/EQT3Q4

**Experimental models: Organisms/strains**		

See Table 2 for strains		

**Oligonucleotides**		

See Table S1 for oligonucleotides		

**Recombinant DNA**		

*pADH99* (Cas9 plasmid)	Nguyen et al.^55^	RRID: Addgene_90979
*pADH110*	Nguyen et al.^55^	RRID: Addgene_90982
*pADH147(HIS-FLP)*	Nguyen et al.^55^	RRID: Addgene_90991
*pCIp10 - γmGFP*	Van Genechten et al.^56^	RRID: Addgene_163119

**Software and algorithms**		

MAFFT-LINSI	v7.526	https://mafft.cbrc.jp/alignment/software/; RRID: SCR_011811
IQ-TREE	v2.0	http://www.iqtree.org/; RRID: SCR_017254
ETE4 Toolkit	v4.4.0	https://etetoolkit.org/
Adobe Photoshop Adobe Photoshop (CS6, Extended)	v26.11 v13.0	https://www.adobe.com/products/photoshop.html; RRID: SCR_014199
Clustal Omega	v1.2.4	http://www.clustal.org/omega/; RRID: SCR_001591
HOLE	v2.2.005	http://www.holeprogram.org/
Visual Molecular Dynamics (VMD)	v1.9.3	https://www.ks.uiuc.edu/Research/vmd/; RRID: SCR_001820
AutoDock Vina	v1.2.0	http://vina.scripps.edu/; RRID: SCR_011958
Maestro	v11.2	https://www.schrodinger.com/platform/products/maestro; RRID: SCR_016748
UCSF Chimera	v1.19	https://www.cgl.ucsf.edu/chimera/; RRID: SCR_004097
hmmscan (HMMER)	v3.4	http://hmmer.org/; RRID: SCR_005305
TM-align	v20220412	https://zhanggroup.org/TM-align/; RRID: SCR_024390
PyMOL (Schrödinger, LLC)	v 3.1.6.1	https://pymol.org/2/; RRID: SCR_000305
NetPhos	v3.1 b	https://services.healthtech.dtu.dk/service.php?NetPhos-3.1; RRID: SCR_017975
GraphPad Prism	v8.0.1	https://www.graphpad.com/; RRID: SCR_002798
trimAl	v1.5.0.	https://trimal.readthedocs.io/en/v1.5.0/; RRID: SCR_017334
LAS AF (Leica Application Suite)	v1.4.1	https://www.leica-microsystems.com/products/microscope-software/las-af-ls/; RRID: SCR_013673
GeneSys	v3.3.0	https://www.cedara.com/products/genesys-software/; RRID: SCR_015770
ImageJ	v1.53 k	https://imagej.nih.gov/ij/; RRID: SCR_003070
QuantaSmart (PerkinElmer)	v5.01	https://www.perkinelmer.com/
PyRx	v0.8	https://pyrx.sourceforge.io; RRID: SCR_018548
Microsoft Excel	V2601	https://www.microsoft.com/en-gb/; RRID: SCR_016137

**Other**		

G:BOX Chemi XX9 — imaging system (gel/blot documentation system)	Syngene	15843280
Leica DM5000B microscope	Leica	11888081 BZ00
Tri-Carb 4810TR Liquid Scintillation Analyzer	PerkinElmer	A481000
UniProtKB	Protein sequences and functional annotations	http://www.uniprot.org/help/uniprotkb; RRID: SCR_004426
AlphaFold Protein Structure Database	Predicted 3D protein structures	https://www.alphafold.ebi.ac.uk/; RRID: SCR_023662
PubChem	Chemical compounds and bioactivity data	https://pubchem.ncbi.nlm.nih.gov/; RRID: SCR_004284
National Center for Biotechnology Information (NCBI)	Genomic, protein, and biomedical databases	https://www.ncbi.nlm.nih.gov/; RRID: SCR_006472

### Experimental model and study participant details

All *C. albicans* strains generated in this study (listed in Table 2) were derived from the WT clinical isolate *C. albicans* SC5314. WT and generated strains were maintained at 30 °C on solid yeast extract-peptone-dextrose (YPD) plates, containing 1% (w/v) yeast extract, 1% (w/v) peptone, 2% (w/v) glucose, and 2% (w/v) agar or in liquid YPD medium with agitation at 200 revolutions per minute (rpm). Strains generated using the CRISPR-Cas9 system were selected on YPD solid medium supplemented with 200 μg/mL nourseothricin. For CRISPR-Cas9 cassette removal, cells were cultured in liquid YP medium containing yeast extract (1% w/v), peptone (1% w/v), and 3% (w/v) maltose at 30 °C and 200 rpm.

For specific assays, cells were grown at 30 °C with shaking at 200 rpm in either SM medium (0.67% w/v YNB with ammonium sulphate) or SC medium (0.67% w/v YNB with ammonium sulphate, 0.2% w/v Kaiser SC mixture) supplemented with the indicated carbon source: glucose (0.2% w/v), acetic acid (0.1% v/v, pH 6.0), lactic acid (0.1% v/v, pH 5.0), succinic acid (0.1% w/v, pH 5.0), butyric acid (0.1% v/v, pH 5.0), formic acid (0.1% v/v, pH 5.0), pyruvic acid (0.1% w/v, pH 6.0), malic acid (0.1% w/v, pH 6.0), or citric acid (0.1% w/v, pH 6.0). The pH of the media containing carboxylic acids was adjusted with a 5 M sodium hydroxide (NaOH) solution.

### Method details

#### *In-silico* analysis

##### Protein sequence alignments and phylogenetic construction

Entries containing the PFAM annotation Gpr1_Fun34_YaaH (PF01184) were collected from UniProtKB using the UniProt API. The sequences were aligned using MAFFT-L-INS-i^57^ and positions with >90% gaps were removed from the alignment with trimAl.^58^ Trees were constructed using IQ-TREE (v2.0)^59^ with model selection on auto (selected model: Q.pfam+G4) and 1,000 iterations of ultrafast bootstrap. Manual inspection was performed for the fungal trees and almost identical branches (representing the same sequence from different strains of the same species or different protein isoforms) were removed. Trees were visualized using ETE4 (https://github.com/etetoolkit/ete4)^60^ and Adobe Photoshop.

The identification of the entire conserved amino acid residues of the AceTr family was performed using Clustal Omega^61^ (www.clustal.org). The protein sequences of the following AceTr members were included in the multiple sequence alignment: *S. cerevisiae* Ato1, Ato2, Ato3; *C. albicans* Ato1, Ato2, Ato3, Ato4, Ato5, Ato6, Ato7, Ato8; *C. glabrata* Ato1, Ato2, Ato3; and *C. auris* Ato1, Ato2, Ato3 (Table 1).

##### Three-dimensional structure and pore radius predictions

The 3D structures of Ato proteins from *S. cerevisiae*, *C. albicans*, *C. glabrata* and *C. auris* were obtained from the AlphaFold Protein Structure Database (Table 1).^48^^,^^62^ These structures were used to predict the radius along the pore using the HOLE program (2.2.005 Linux).^63^ The simple van der Waals radii file provided with HOLE program was used for pore prediction in each Ato protein. The resulting predictions were visualized together with the 3D protein structures using the Visual Molecular Dynamics software (VMD, 1.9.3).^64^ Pore radius profiles of the Ato proteins were plotted using Microsoft Excel (version 2601), with the x-axis representing the position along the pore axis.^65^

In addition, the average predicted Local Distance Difference Test (pLDDT) scores obtained from AlphaFold, which reflect per-residue structural confidence on a 0–100 scale, indicated high confidence 3D structure predictions for all Ato proteins, ranging from 77.8 to 86.0 (Table S2). Notably, residues located within the pore and constriction regions exhibited very high pLDDT scores, supporting the reliability of these structural predictions.

##### Molecular docking

Molecular docking simulations of acetate and lactate substrates were performed using AutoDock Vina through the PyRx virtual screening tool,^66^ as previously described.^42^ The ligand structures of acetate and lactate were obtained from PubChem (https://pubchem.ncbi.nlm.nih.gov/). The simulated ligand-protein interactions were visualized and analyzed by Maestro (v11.2) and UCSF Chimera, as a graphical user interface for AutoDock Vina.^42^ The dissociated forms of each carboxylic acid were used in the docking studies.

##### Domain annotation

Domains were predicted using Hmmscan (HMMER 3.4, Aug 2023; http://hmmer.org/) for the HMM-profiles of interest (PF01184: Gpr1_Fun34_YaaH and PF01300: Sua5_yciO_yrdC). Hits with a bit-score > 25 for PF01300 and > 0 for PF01184 (increasing sensitivity to predict some of the more divergent metazoan domains) were considered. Domains were visualized next to the trees using ETE4.

##### Structural superimposition and visualization

The crystal structure for SatP^33^ (5ZUG) and an AlphaFold 2.0 predicted structure for A0A3B3D9A3^48^ were used. Structures were superimposed using the TM-align webserver (https://zhanggroup.org/TM-align/) to calculate TM-score and RMSD values. For visualization, PyMOL (Version 3.0 Schrödinger, LLC) was used. Structures were superimposed using the PyMOL super command and figures were finalized using Adobe Photoshop.

##### Phosphorylation sites prediction

Phosphorylation site prediction was performed using NetPhos-3.1b (https://services.healthtech.dtu.dk/services/NetPhos-3.1/).^67^^,^^68^ This tool uses multiple neural networks to make predictions.

#### Genetic manipulation of *C. albicans* using the CRISPR-Cas9 system for generation of GFP fusions and gene deletions

*GFP* fusions at the 3’ terminal of *CaATO* genes (Table 2) were performed using the CRISPR-Cas9 system following an adapted version of the protocol from Hernday lab.^55^ In summary, each gene's customized gRNA expression cassette (Fragment C) was generated by assembling fragments A and B using cloning-free stitching PCR (with primers Hernday-AHO1237 and Hernday-AHO1236). Fragment A was amplified from pADH110 (with Hernday-AHO1096 and Hernday-AHO1098 primers), while fragment B was amplified from pADH147 (with Hernday-AHO1097 and Hernday-gRNA-GOI-GFP primers). The Cas9 cassette was obtained by digestion of the pADH99 plasmid with the *MssI* restriction enzyme. In a single transformation, the Cas9 and gRNA cassettes were co-transformed along with a specific donor's DNA-GFP (linear DNA fragment), which was amplified from the template CIp10 - γmGFP (with primers Hernday-dDNA-GOI-GFP-FW and Hernday-dDNA-GOI-GFP-RV).^56^ Positive *C. albicans* transformants were selected on YPD medium supplemented with nourseothricin and verified by colony PCR (with GOI-FW-check and GOI-RV-check or Inside-GFP-Check-RV primers). After confirming GFP integration, the NAT marker, Cas9, and gRNA expression cassettes were removed by inducing the maltose-inducible FLP recombinase system in YP medium with 3% maltose, followed by overnight incubation at 30 °C. To verify the effective removal of CRISPR components, cells were streaked onto YPD agar to isolate single colonies. Single colonies were then tested on both YPD and YPD supplemented with nourseothricin. Next, the genotypes of all generated strains were verified by colony PCR and confirmed through sequencing.

*C. albicans* Ato-GFP strains, carrying individual *ato* deletions (Table 2), were generated using a modified transient CRISPR-Cas9 approach.^69^ All sgRNA expression cassettes were constructed through a three-step PCR process using the pV1093 plasmid as a template. The steps included (i) synthesizing the SNR52 promoter (with oligonucleotides: SNR52/F and SNR52/R-GOI), (ii) generating the sgRNA scaffold (with primers: sgRNA/F-GOI, and sgRNA/R) and (iii) fusing the SNR52 promoter with sgRNA scaffold cassettes (with primers: SNR52/N and sgRNA/N). The Cas9 expression cassette was amplified from the pV1093 plasmid via PCR using the CaCas9/F and CaCas9/R primers. Similarly, repair templates specific to the desired gene were generated through PCR using the primers NAT-GOI-repair/F and NAT-GOI-repair/R. *C. albicans* cells were simultaneously transformed with the three constructed cassettes using the classical lithium acetate method.^70^ Positive *C. albicans* transformants were selected in YPD supplemented with nourseothricin and, subsequently, validated through colony PCR (using the oligonucleotides GOI-FW-check and GOI-RV-check). All primers used in this study are listed in Table S1**.**

#### Epifluorescence microscopy

*C. albicans* cells expressing Ato-GFP fusion proteins were grown in SM medium supplemented with 0.2% glucose until the exponential phase at 30 °C and 200 rpm. Cells were then washed and transferred to SM medium containing either acetic acid (0.1% v/v, pH 6.0), lactic acid (0.1% v/v, pH 5.0), or no added carbon source, and incubated at 30 °C, and 200 rpm. Samples were collected at 0, 5, and 24 h after derepression for analyses by epifluorescence microscopy and subsequent protein extraction (see section Western Blot and quantification analyses). Moreover, for further investigation, cells expressing Ato-GFP fusion proteins were grown directly in SC medium containing different carboxylic acids: formic acid (0.1% v/v, pH 5.0), butyric acid (0.1% v/v, pH 5.0), succinic acid (0.1% w/v, pH 5.0), pyruvic acid (0.1% w/v, pH 6.0), malic acid (0.1% w/v, pH 6.0), citric acid (0.1% w/v, pH 6.0) and 0.2% (w/v) glucose at 30 °C and 200 rpm. Cells were visualized by fluorescence microscopy after 16, 18, 20, 24, and 40 h of growth in the specified medium. The time point t≈16 h corresponded to the beginning of exponential phase (0.4 < OD_640 nm_ < 0.6). For microscopy analysis, 1 mL of each culture was harvested, cells were concentrated by centrifugation and mounted on microscope slides. Cells were visualized using a Leica DM5000B fluorescence microscope with appropriate filters. Images were captured with a Leica DFC 350FX R2 digital camera using LAS AF V1.4.1 software (Leica). Microscopy images were organized and assembled into final figures using Adobe Photoshop (CS6, Extended v13.0). Microscopy data presented in the main figures were derived from three independent experiments, while data presented in the supplementary figures represent two independent experiments.

#### Western Blot and quantification analyses

Yeast cells were grown under the conditions described in the preceding section. After 5 h of induction, cells were harvested at OD_640__nm_ = 1.5, and total protein extracts were prepared using the NaOH/TCA method.^71^ Briefly, cells were harvested by centrifugation (max. rpm, 1 minute (min), 4 °C) and the pellet was resuspended in 500 μL of cold water. After addition of 50 μL of NaOH (1.85 M), samples were incubated on ice for 10 min. Then, 50 μL of trichloroacetic acid (TCA, 50%) was added and the samples were incubated for an additional 10 min on ice. Protein precipitates were collected by centrifugation (max. rpm, 15 min, 4 °C). The pellets were dissolved in 50 μL of loading buffer (33.3 mM Tris-hydrochloride pH 6.8; 1.33 mM ethylenediaminetetraacetic acid (EDTA); 1.33% sodium dodecyl sulphate (SDS); 6.66% glycerol; 0.02% bromophenol blue and 2% beta-mercaptoethanol) and heated at 37 °C for 10 min. Each sample (10 μL) was loaded onto a 10% SDS-polyacrylamide gel. Following electrophoresis, the separated proteins were transferred onto nitrocellulose membranes (GE Healthcare Life Sciences. For transfer assessment and loading controls, the membranes were stained with a Ponceau S solution (0.1% Ponceau (w/v); 0.5% glacial acetic acid (v/v)). Following washing and blocking steps, the membranes were probed with the primary antibody anti-GFP (monoclonal, mouse IgG1κ, clones 7.1 and 13.1, Roche, 11814460001) used at 1:3000 dilution. The antimouse-IgG (whole molecule)-peroxidase produced in rabbit (A9044, Sigma) was used as a secondary antibody at 1:100000 dilution. The signal was detected by enhanced chemiluminescence using the WesternBright ECL HRP substrate (advansta). All images were captured using a Syngene G: Box Chemi XX9 image documentation system and GeneSys software. GFP signal intensity was quantified using ImageJ software (version 1.53k) and normalized to the Ponceau S staining. The data are represented in arbitrary units (A.U.) as the mean of at least three independent experiments (n≥3) (Figure S8). The error bars represent the standard error of the mean (SEM) (Prism 8.0; GraphPad software, version 8.0.1).

#### Cell staining with DAPI

*C. albicans* Ato2-GFP and Ato2-GFP *ato1Δ/Δ* strains were grown in a SM medium (0.2% glucose) to mid-log phase (OD_640__nm_ = 0.5). Cells were harvested, washed twice with deionized water and transferred to SM media containing acetic acid (0.1% v/v, pH 6.0). After 5 h of derepression, 1 ml of cells was collected, and resuspended in 1 ml 3.7% paraformaldehyde (PFA) then incubate 1 h at room temperature with shaking at 200 rpm. After fixation, cells were washed three times with phosphate-buffered saline (PBS) and resuspended in 1 ml PBS. DAPI nuclear stain was added to a final concentration of 2 μg/mL, and cells were incubated at room temperature for 10 min with shaking at 200 rpm. Following two additional washes with PBS, cells were visualized by fluorescence microscopy.

#### Transport assays with radiolabeled substrates

*C. albicans* WT, Ato1-GFP, *ato1Δ/Δ* and *ato1-10Δ/Δ* strains were grown in SM medium supplemented with 0.2% glucose until reaching an OD_640 nm_ = 0.5. Cells were then washed twice in deionized water and transferred to SM medium containing acetic acid (0.1% v/v, pH 6.0). After 5 h of derepression, cells were harvested, washed twice with deionized water and resuspended in water to a final concentration of 20–40 mg (dry weight)/mL. For each reaction, 30 μL of the cell suspension was mixed with 60 μL of 100 mM potassium phosphate buffer (pH 6.0) and incubated for 5 min at 30 °C.

Transport assays in the presence of inhibitors were carried out for 30 seconds (s) with 5 μL of [1-^14^C] acetate (final concentration 0.1 mM, 3000 disintegrations per minute (dpm); sodium salt, 55.2 mCi/mmol, PerkinElmer) and 5 μL of non-radioactive (unlabeled) acids (final concentration 10 mM) including: acetate, lactate, pyruvate, formate, butyrate, succinate, malate and citrate. Transport assays with protonophores or ionophores were performed by adding 5 μL of [1-^14^C]-acetate (final concentration 0.1 mM, 3000 dpm; sodium salt, 55.2 mCi/mmol, PerkinElmer) or [1-^14^C]-lactate (final concentration 2 mM, 1000 dpm sodium salt, 154.8 mCi/mmol, Perkin Elmer) to cells in the presence of CCCP and valinomycin at a final concentration of 50 μM, with a total reaction time of 30 s. Transport assays of lactate were carried out for 15 s with 10 μL of [1-^14^C]-lactate (sodium salt, 154.8 mCi/mmol, Perkin Elmer) at final concentrations ranging from 1 to 24 mM, with adjusted specific activities (200-1000 dpm). For acetate uptake assays, reactions were initiated by adding 10 μL of [1-^14^C]-acetate (final concentration 4 mM, 500 dpm) and incubated for 15 s.

For all assays, reactions were stopped by adding 100 μL of ice-cold acetic acid (pH 6.0) or lactic acid (pH 6.0) at final concentration 200 mM. Following incubation, the reaction mixtures were kept on ice, centrifuged at 13000 rpm. Then the pellet was washed with 1 mL ice-cold deionized water and resuspended in 1 mL of scintillation fluid (Opti-phase HiSafe II). Radioactivity was quantified using a Packard Tri-Carb 2200 CA liquid scintillation counter. Each reaction was prepared duplicate, and data represents standard deviation (SD) from at least three independent experiments (n=6).

### Quantification and statistical analysis

Statistical analyses were performed using GraphPad Software, Prism 8. Data distributions were tested for normality. For western blot assays quantification of the protein levels were done using ImageJ software (version 1.53k) and analysed using unpaired t-test (two-tailed). For transport assays datasets that failed to meet normality assumptions, group differences were assessed using the Kruskal–Wallis test and Mann–Whitney U test. For normally distributed data with unequal variances, Welch’s one-way analysis of variance (ANOVA) was used, followed by Dunnett’s T3 post hoc test for multiple comparisons. Statistical significance was set at *p* < 0.05.
