# Supplementary material for: Expansion, functional diversification, and gene fusion events in the Ato protein family
Source: iScience. 2026 Jun 22;29(7):116467. doi: 10.1016/j.isci.2026.116467 (PMC13316224; doi:10.1016/j.isci.2026.116467)
Supplement: Document S1. Figures S1–S12 and Tables S1–S3 [file mmc1.pdf]

iScience, Volume *29*

Supplemental information

Expansion, functional diversification, and gene fusion events in the Ato protein family

Faezeh Ghasemi, Patrícia Ataíde, Cláudia Barata-Antunes, Yiannis Pyrris, João Alves, Vitor Fernandes, Rosana Alves, Alexandra Gomes-Gonçalves, Margarida Casal, Wouter Van Genechten, Jana Nysten, Alistair J.P. Brown, Patrick Van Dijck, Alexandros A. Pittis, George Diallinas, Isabel Soares-Silva, and Sandra Paiva

Table S1. List of oligonucleotides used in this work, related to STAR★METHODS.

Name	Sequence
Hernday-AHO1096	GACGGCACGGCCACGCGTTTAAACCGCC
Hernday-AHO1098	CAAATTAAAAATAGTTTACGCAAG
Hernday-AHO1097	CCCGCCAGGCGCTGGGGTTTAAACACCG
Hernday-AHO1237	AGGTGATGCTGAAGCTATTGAAG
Hernday-AHO1236	TAAAGCTGCCACAAGAGGTATTTC
Hernday-gRNA-ATO1-GFP	CGTAAACTATTTTTAATTTGGGATCTTGTAAATCTGGTAAGTTTTAGAGCTAGAAATAG
Hernday-gRNA-ATO2-GFP	CGTAAACTATTTTTAATTTGTCTGAAGTAGGAGTTTGTTGGTTTTAGAGCTAGAAATAG
Hernday-gRNA-ATO3-GFP	CGTAAACTATTTTTAATTTGGCAATGGAATAGAAACAGGTGTTTTAGAGCTAGAAATAG
Hernday-gRNA-ATO4-GFP	CGTAAACTATTTTTAATTTGTCTGGTAAAGGAATTTCTCTGTTTTAGAGCTAGAAATAG
Hernday-gRNA-ATO5-GFP	CGTAAACTATTTTTAATTTGTCTGGTAAGGGAATTTCTTTGTTTTAGAGCTAGAAATAG
Hernday-gRNA-ATO6-GFP	CGTAAACTATTTTTAATTTGCCAAATTGAGGGACTGGAAGGTTTTAGAGCTAGAAATAG
Hernday-gRNA-ATO7-GFP	CGTAAACTATTTTTAATTTGTTTTTGTTACCCCCAAGATGGTTTTAGAGCTAGAAATAG
Hernday-gRNA-ATO8-GFP	CGTAAACTATTTTTAATTTGTTTGCCTCACTTCCTGGCAAGTTTTAGAGCTAGAAATAG
Hernday-gRNA-ATO9-GFP	CGTAAACTATTTTTAATTTGGGGTAATTGTTGTATAATCGGTTTTAGAGCTAGAAATAG
Hernday-gRNA-ATO10-GFP	CGTAAACTATTTTTAATTTGCTTGGTTATCAAGACATCACGTTTTAGAGCTAGAAATAG
SNR52/F	AAGAAAGAAAGAAAACCAGGAGTGAA
SNR52/R-ATO1	CCAAATGCTTCAACTAAATCCAAATTAAAAATAGTTTACGCAAGTC
SNR52/R-ATO2	CCGAAAGCAGCCATCAATTCCAAATTAAAAATAGTTTACGCAAGTC
SNR52/R-ATO3	CCGAAGGCAGCCATCAAGTCCAAATTAAAAATAGTTTACGCAAGTC
sgRNA/F-ATO1	GATTTAGTTGAAGCATTTGGGTTTTAGAGCTAGAAATAGCAAGTTAAA
sgRNA/F-ATO2	GAATTGATGGCTGCTTTCGGGTTTTAGAGCTAGAAATAGCAAGTTAAA
sgRNA/F-ATO3	GACTTGATGGCTGCCTTCGGGTTTTAGAGCTAGAAATAGCAAGTTAAA
sgRNA/R	ACAAATATTTAAACTCGGGACCTGG
SNR52/N	GCGGCCGCAAGTGATTAGACT
sgRNA/N	GCAGCTCAGTGATTAAGAGTAAAGATGG
CaCas9/F	ATCTCATTAGATTTGGAACTTGTGGGTT
CaCas9/R	TTCGAGCGTCCCAAAACCTTCT
Hernday-dDNA-ATO1-GFP-FW	GGTATTGCTAATCCTCAAAATAGTTATATTACTGTTAAAGCTATTCCATTACCAGATTTACAAGATCCAACAAGAAAAAATAAAGGTGCTGGCGCAGGTGCTATGTCTAAAGGTGAAGAATT

Hernday-dDNA-ATO1-GFP-RV	TTGATTGATTGATTGATTGATTGATTGATTACTTGGCCAATTATTTTATTTGTACAATTCATCCA
Hernday-dDNA-ATO2-GFP-FW	TCACTGCTATCATCGCTTGGTATGTTGCTTTAGCAGGTACAGCCACTACAACAAACTCCTACTTCAGACCAATTTCCATTCCAATGCCAGGAAATGTTGCTTTTAAAAACGGTGCTGGCGCAGGTGCTATGTCTAAAGGTGAAGAATT
Hernday-dDNA-ATO2-GFP-RV	AGCATTAAAATTAGATAAGTAGCAACAACTGAAAGAGTTTTGCCATTATTTGTACAATTCATCCA
Hernday-dDNA-ATO3-GFP-FW	GTGGAATGCCTTAGCCGGTACTGCTACTCCAACCAACTCTTACTTTCAACCTGTTTCTATTCCATTGCCAGGTAACGTTGTTTTCAAGAAAGGTGCTGGCGCAGGTGCTATGTCTAAAGGTGAAGAATT
Hernday-dDNA-ATO3-GFP-RV	GATAGCCATGGTGAATGACAATAAAATGCAGCTAGACAGATATTTTTATTTGTACAATTCATCCA
Hernday-dDNA-ATO4-GFP-FW	GAAGCTTTTGCTGGGGTAGCTAATAGACAAAACTCTTACATGGTTCCCCGTGAGATACCTTTACCAGATCTCTCAGTCTGGCTTAAACGTAAAAAAGCTGTCAGCAAAACATCAAATGGTGCTGGCGCAGGTGCTATGTCTAAAGGTGAAGAATT
Hernday-dDNA-ATO4-GFP-RV	AATACTGAATCTTTAGGGTTATTGTAGCCGGGTTCCGTTTTTGAGTTATTTGTACAATTCATCCA
Hernday-dDNA-ATO5-GFP-FW	GAAGCTTTCGCTGGTGTGGCCAACACACATAATTCATATTTGGTTCCCAAGGAGATACCCTTACCAGATCTTTCACTTTGGTTTAAACGTAAAAAGTCGGAAAATCAACCATCAGAGGGTGCTGGCGCAGGTGCTATGTCTAAAGGTGAAGAATT
Hernday-dDNA-ATO5-GFP-RV	AAATGTTTCCAGAACAAGTTACAAACAAAAATATAACGTTATACTTTATTTGTACAATTCATCCA
Hernday-dDNA-ATO6-GFP-FW	ATTTCTGGTATGGCAGATAAATTCAATTCTTATTTCACAGTTCATCCATTACCTGTTCCTCAATTTGGAAAGAAAAATGGTGCTGGCGCAGGTGCTATGTCTAAAGGTGAAGAATT
Hernday-dDNA-ATO6-GFP-RV	CATGAATAATAGAATTCTAGGATGCACAGCATAGCTAGTTTAGATTTATTTGTACAATTCATCCA
Hernday-dDNA-ATO7-GFP-FW	GCTGATAGATATAATTCTTATTTTACTATCAATCCAGTTCCAATTCCACATCTTGGGGGTAACAAAAAACAAAAAGAGATGAGAGGTGCTGGCGCAGGTGCTATGTCTAAAGGTGAAGAATT
Hernday-dDNA-ATO7-GFP-RV	AGACAAGTTGACCTCCCCCTTTTTCTGTAGAACTCAGATCTCATCTTATTTGTACAATTCATCCA
Hernday-dDNA-ATO8-GFP-FW	TTATTGGCAACAAAACAGAACTCTTACTTTACACTTTCTGTGATTTCATTGCCAGGAAGTGAGGCAAAACAAAAGAAAGGTGCTGGCGCAGGTGCTATGTCTAAAGGTGAAGAATT
Hernday-dDNA-ATO8-GFP-RV	AATGAAAGAAATCCAAATAAACCGTTTTCCAGCTCTTAACCTTTGTTATTTGTACAATTCATCCA
Hernday-dDNA-ATO9-GFP-FW	TAGCTGGTGTTTGGGAATTTTTTGTGGGTAATTGTTGTATAATCGTTGCATTCGACTCAAATAAAGAACAGATTGCATTACCAATTGGTGCTGGCGCAGGTGCTATGTCTAAAGGTGAAGAATT
Hernday-dDNA-ATO9-GFP-RV	CTAATTTACGCTAAGGTGTTAGCTTATTGGACCAACTACTTTAAATTATTTGTACAATTCATCCA
Hernday-dDNA-ATO10-GFP-FW	AATTCATATTTTGTCATTCCTATCACTTGGTTATCAAGACATCACGGTAAGGGTGCTGGCGCAGGTGCTATGTCTAAAGGTGAAGAATT
Hernday-dDNA-ATO10-GFP-RV	AAGTTTGTTGTCTTCCCCCTTTGATTGTAGTTTTCCACTAACTTCTTATTTGTACAATTCATCCA
NAT-ATO1-repair/F	ACACTACAATAAACTTTTAACAACACACATAAATAACTAACTACTACAACTACAACTACAACCACTACACTTATCAAATCAGTCTAATCACTTGCGGCCGC
NAT-ATO1-repair/R	TCATCATTAAAAAAAAAAATCAATACCAGTATTGATTGATTGATTGATTGATTGATTGATTGATTACTTGGCCAATTATTGGACCACCTTTGATTGTAAATAG
NAT-ATO2-repair/F	CCAAATTGCTGTTCATTTGTTTTAGCTATTATTTTGTTCGTTCTACGAAGAATCTTATTATTGATCTACTGTTAAAGAATAGTCTAATCACTTGCGGCCGC
NAT-ATO2-repair/R	TTATAGTTGAATCGTACAATTGAACAATCAAATTAAGCATTAAAATTAGATAAGTAGCAACAACTGAAAGAGTTTTGCCAGGACCACCTTTGATTGTAAATAG

NAT-ATO3-repair/F	TTCGAAATAGAAAAAAACTGTTTCTTTTATATAAAGTTCTACTATCTATATCCA ATATCGATAAATAAAAGGAAACAACTAGTCTAATCACTTGCGGCCGC
NAT-ATO3-repair/R	AATTATAGTAGCAATAAAGAAAAAAAAAAATCAAATGATAGCCATGGTGAATGA CAATAAAATGCAGCTAGACAGATATTTGGACCACCTTTGATTGTAAATAG
ATO1-FW-Check	ACAACTACAACTACAACCACTACACT
ATO1-RV-Check	ACACACACACACACACAGACCCA
ATO2-FW-Check	TCCAAGTTGGCCAAATTGCTGT
ATO2-RV-Check	GCAACAACTGAAAGAGTTTTGCCA
ATO3-FW-Check	TGATGTTTAGTTTTCACACCCCCA
ATO3-RV-Check	GCCATGGTGAATGACAATAAAATGCA
ATO4-FW-Check	GGCTATAAAAGGCCAACCAT
ATO4-RV-Check	TCGTCGTCAGGTAATTGCAG
ATO5-FW-Check	GGATGAACAGCCTTATTGTT
ATO5-RV-Check	GTTCGATCATCAACCTTCAC
ATO6-FW-Check	TTGTAGTTTCACTCAGGTTT
ATO6-RV-Check	TCCATGAGTTACCTCCACTG
ATO7-FW-Check	ACTCAGACCATCCATCCTCCTA
ATO7-RV-Check	ACCAGACAAGTTGACCTCCCCCT
ATO8-FW-Check	GTTACAATTGCAAACTGCTT
ATO8-RV-Check	AAGTAGTCGTGCATGTTTTC
ATO9-FW-Check	CCAATACCACTCTTTAATGT
ATO9-RV-Check	GGAAGGACGTGAAACATGAG
ATO10-FW-Check	CGTTCTAAATCTGTGTAGGC
ATO10-RV-Check	GCTAATTATGAGATAGCTTC
Inside-GFP-Check-RV	GTAATACCAGCAGCAGTAAC

Table S2. Average pLDDT values for AlphaFold-predicted Ato proteins from *C. albicans* (CaAto1-8), *C. glabrata* (CgAto1-3), *C. auris* (CauAto1-3) *and S. cerevisiae* (ScAto1), related to Figure 2 and Figure S3.

Protein	Average pLDDT	Confidence level
CaAto1	80.06	High
CaAto2	86	High
CaAto3	85.88	High
CaAto4	80.94	High
CaAto5	81.19	High
CaAto6	84.94	High
CaAto7	81.06	High
CaAto8	85.94	High
CgAto1	78.5	High
CgAto2	77.81	High
CgAto3	81.31	High
CauAto1	85.38	High
CauAto2	82.5	High
CauAto3	82.44	High
ScAto1	79.5	High

Table S3. Average of the acetate and lactate binding affinity values (kcal/mol) for substrate docking of ScAto1, CaAto1-8, CgAto1-3 and CauAto1-3 calculated with PyRx software, related to Figure 3 and Figure S4.

3D-Protein templates	Average of binding affinities (kcal/mol) at different binding sites						
	Acetate						
	S4			S3	S2	S1	
	a	b	c			a	b
**ScAto1**	-2.6	-3.1	-2.4	-3.1	-2.9	-2.5	
**CaAto1**	-2.4	-2.1		-3	-2.8	-2.4	-2.1
**CaAto2**	-2.6	-2.5	-2.7	-2.9	-3	-2.7	-2.4
**CaAto3**	-2.8	-2.6		-2.9	-3.2	-2.4	
**CaAto4**			-2.8	-2.9	-3		-2.3
**CaAto5**	-2.8			-2.5	-2.9	-2.9	
**CaAto6**	-2.6	-3	-2.1	-2.7	-3	-2.3	-2.9
**CaAto7**		-2.4	-3.2	-2.5	-3		-2.9
**CaAto8**	-2.6	-2.6		-2.8	-2.8	-2.6	
**CgAto1**	-2.4			-2.8	-3.1	-2.5	-2.3
**CgAto2**	-2.5	-2.8	-2.6	-3.2	-3.2	-2.6	-2.2
**CgAto3**	-2.4	-2.9		-3	-2.9	-2.4	-2
**CauAto1**		-2.4		-2.8	-3.2	-2.5	
**CauAto2**	-2.4		-2.3	-3.1	-3.1	-2.5	
**CauAto3**		-2.8		-2.9	-2.9		-2.3

	Average of binding affinities (kcal/mol) at different binding sites						
	Lactate						
	S4			S3	S2	S1	
	a	b	c			a	b
**ScAto1**	-3.3		-3.9	-3.9	-3.2	-3.1	-2.9
**CaAto1**	-3.2	-3.1		-3.5	-3.4	-3.2	-2.8
**CaAto2**	-2.9	-3.1	-3.1	-3.2	-2.9	-3.1	-3.2
**CaAto3**	-3.1	-3.2	-3.3	-3.8	-3.5		-3.2
**CaAto4**			-3.1	-3.4	-4.2		-3.7
**CaAto5**	-3.6			-3.1	-3.9	-3	
**CaAto6**	-3.4		-3	-3.3	-2.5	-3	-3.5
**CaAto7**		-3.1	-3.4	-3.2	-3.7		-4
**CaAto8**	-2.9	-3.5	-3.1		-2.6	-2.8	
**CgAto1**	-3.4		-3.1	-3.5	-3.3	-3.1	-3.1
**CgAto2**	-3.2		-3.8	-3.9	-3.3		-3.2
**CgAto3**	-3.1	-3.3	-3.2	-3.8	-2.6		-2.5
**CauAto1**		-3.5		-3.9	-3.6		-3
**CauAto2**	-3.3		-3.3	-3.8	-3.7		-3
**CauAto3**		-3.1		-3.4	-2.9		-3

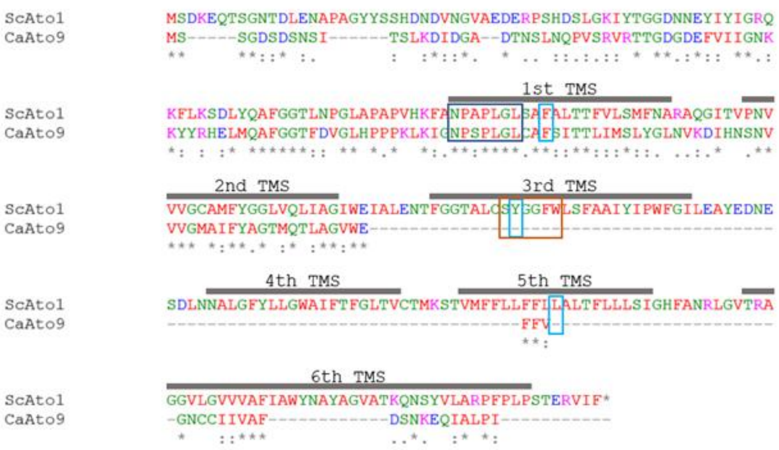

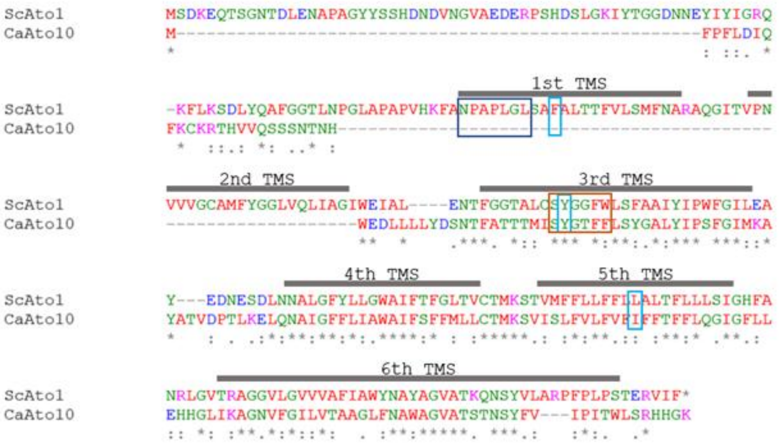

Figure S1. Alignment of Ato9 and Ato10 protein sequences of *C. albicans* with Ato1 of *S. cerevisiae*, related to Figure 1. The ClustalOmega was used to obtain the sequence alignment. The transmembrane regions of Ato1 of *S. cerevisiae* are highlighted in gray. The signature motif, N-P-A-P-L-G-L of the AceTr family is highlighted with dark blue rectangle. The narrowest hydrophobic constriction region (FLY) is represented by light blue rectangles. A second signature motif S-Y[F]-G-F-W located in the third TMS is highlighted in orange.

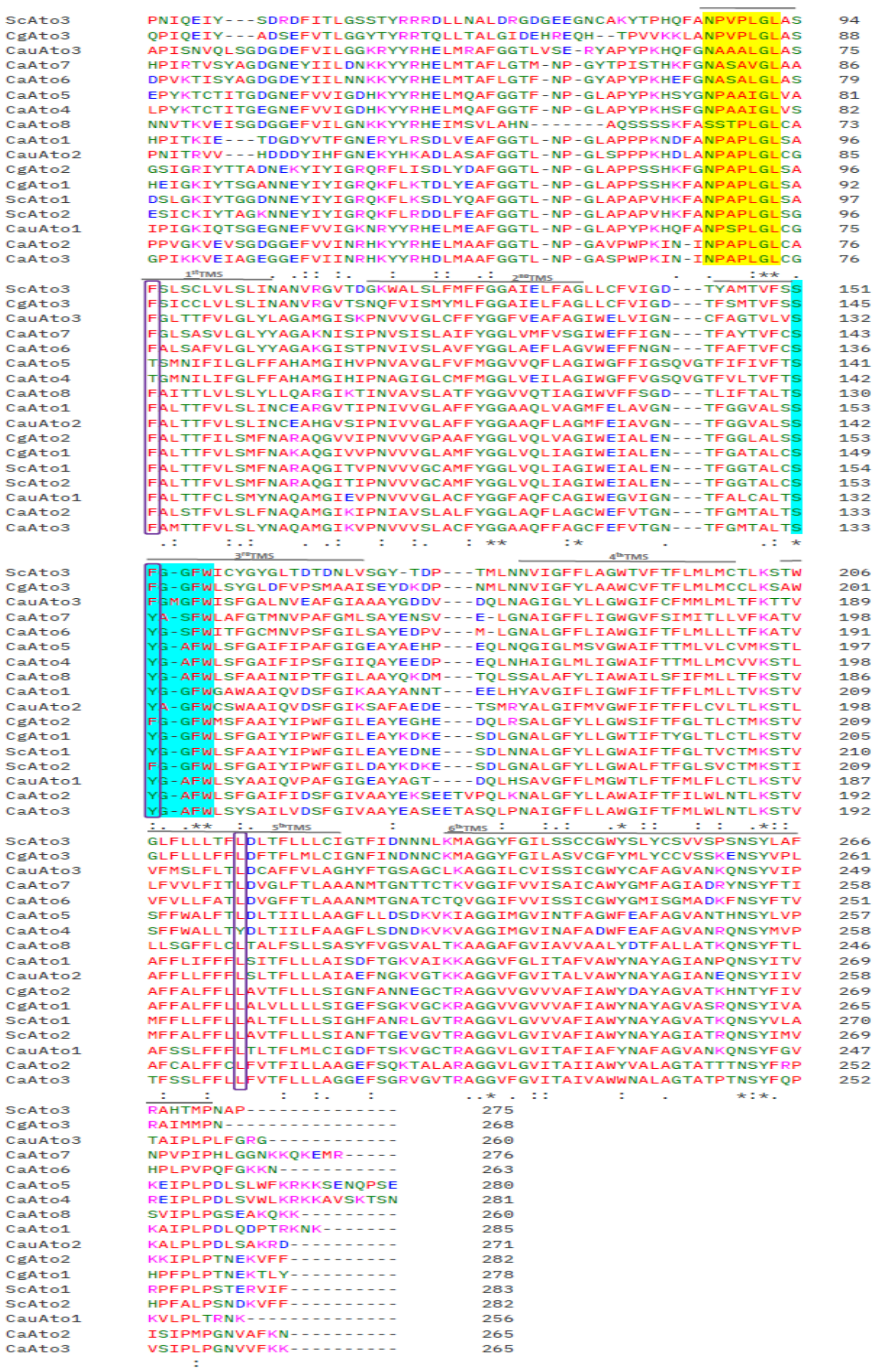

Figure S2. Alignment of Ato homologous sequences from *C. albicans* (CaAto1-8), *C. glabrata* (CgAto1-3), *C. auris* (CauAto1-3) and *S. cerevisiae* (ScAto1-3), related to Figure 1. The ClustalOmega was used to obtain the sequence alignment. The signature motif, N-P-A-P-L-G-L of the AceTr family is highlighted in yellow background. The narrowest hydrophobic constriction region (FLY) is represented by purple rectangles. A second signature motif S-Y[F]-G-F-W located in the third TMS is highlighted in cyan.

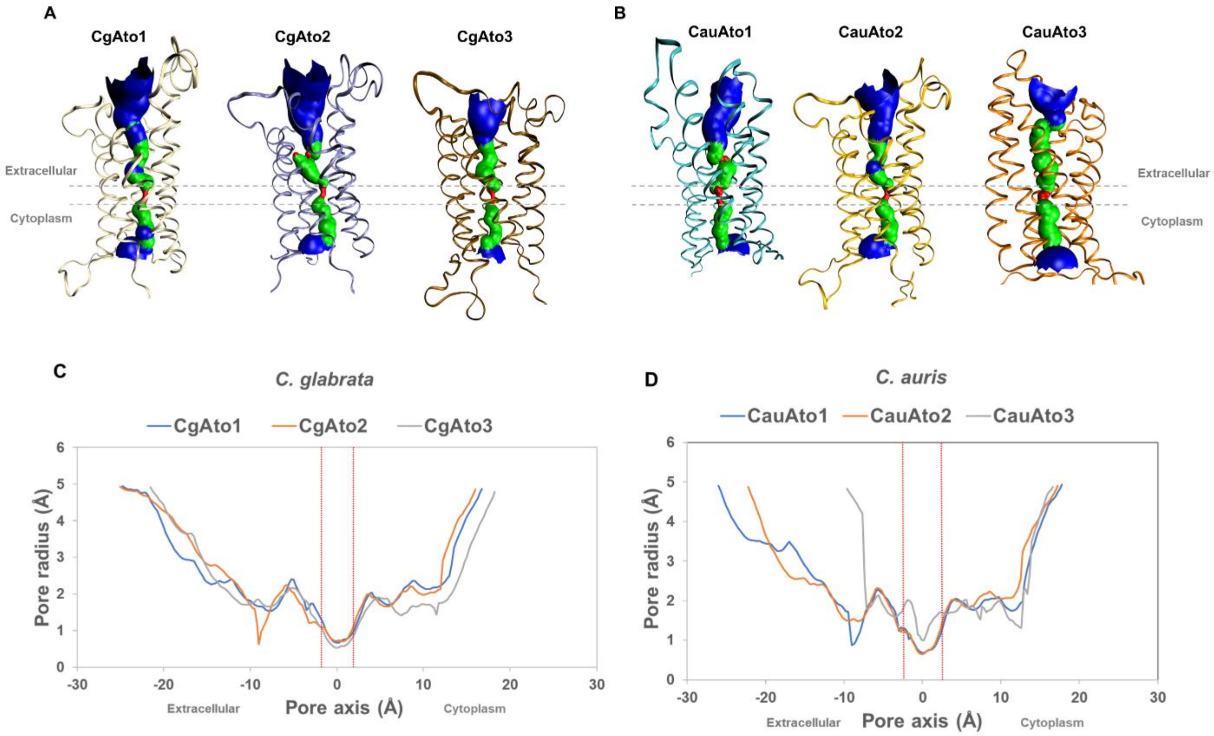

Figure S3. Predicted 3D pore structures and radius profiles along the channel axis in Atos proteins from *C. glabrata*, and *C. auris*, related to Figure 2. A. Pore 3D structure prediction of CgAto1-3. **B.** Pore 3D structure prediction of CauAto1-3. The 3D structures of proteins are shown in Ribbon representation, with a color scheme in which blue represents a larger pore size, green an intermediate pore size, and red a more constricted pore size as predicted by pore analysis. The horizontal dashed grey lines correspond to the constricted site where the pore radius is tight. **C.** Simulations for the pore radius profiles along the channel axis in CgAto1-3. **D.** Simulations for the pore radius profiles along the channel axis in CauAto1-3. The central region of the proteins containing the construction site is indicated by vertical red dashed lines.

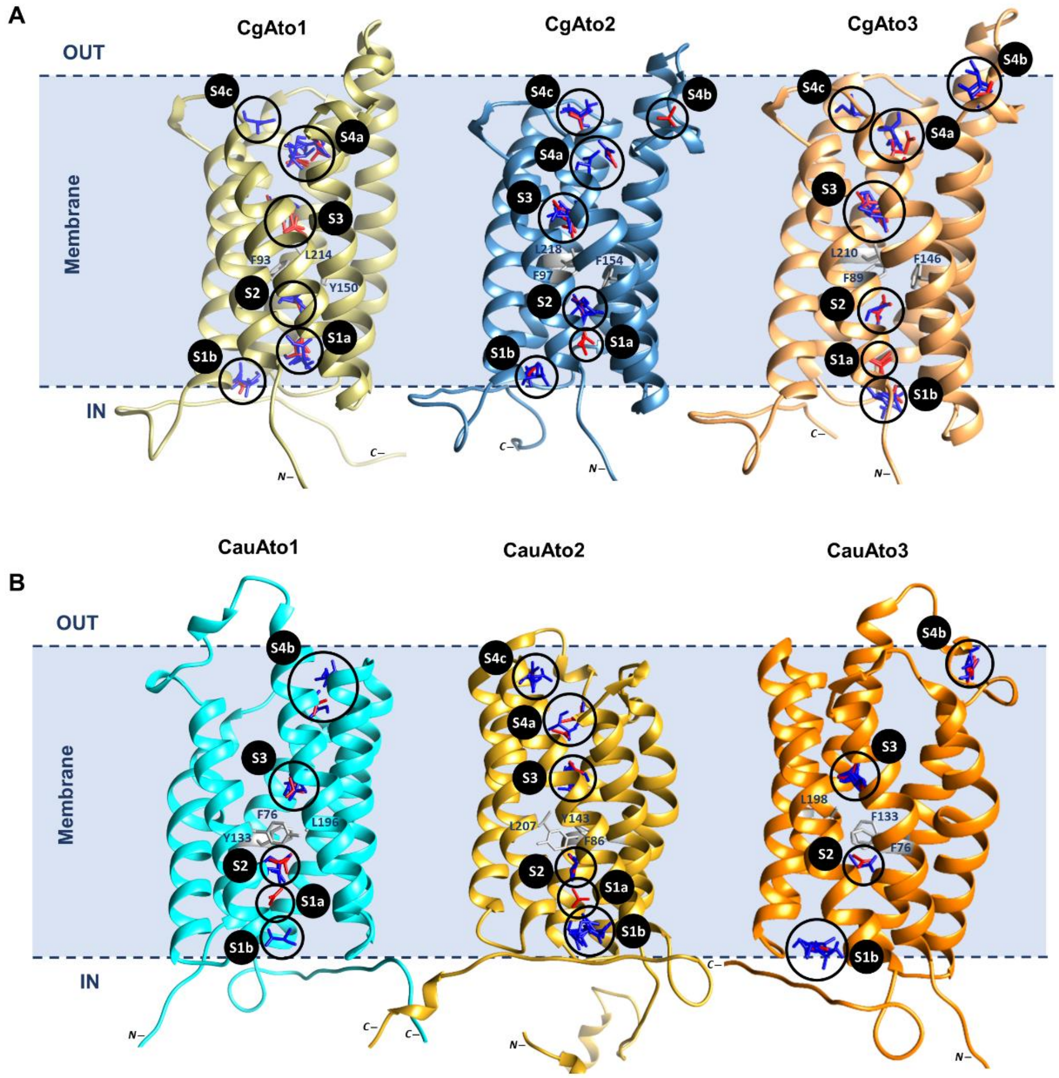

Figure S4. 3D structure and molecular docking, related to Figure 3. A. Molecular docking of CgAto1-3 with acetate and lactate as substrates. **B.** Molecular docking of CauAto1-3 with acetate and lactate as substrates. Predicted binding sites for acetate and lactate were shown with S1 to S4. Site S1 is located at the cytoplasmic vestibule, Sites S2 and S3 are located inside the main pore, and Site S4 is located at the extracellular vestibule. Localization of the N- and C-terminal of the proteins is shown. Acetate and lactate ligands are presented in red and blue respectively.

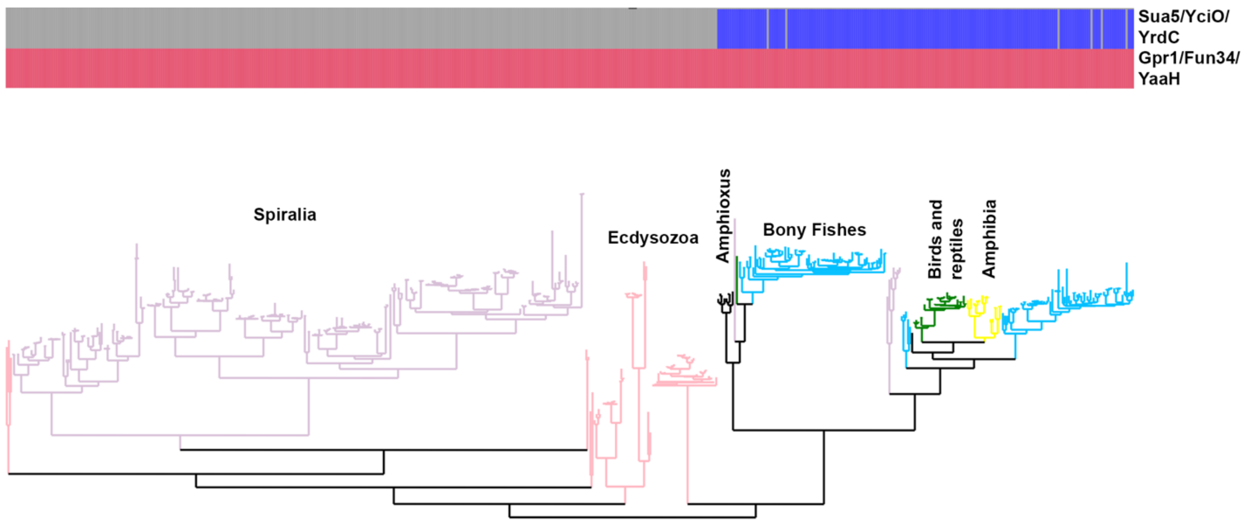

Figure S5. Metazoan Ato phylogeny and structure, related to Figure 7. Phylogeny and domain composition of metazoan Ato proteins. Domain presence is indicated in blue or red and absence in grey. Tree branches are colored according to taxonomic classification. Two major clades are observed: a protostomian clade and a predominantly vertebrate clade.

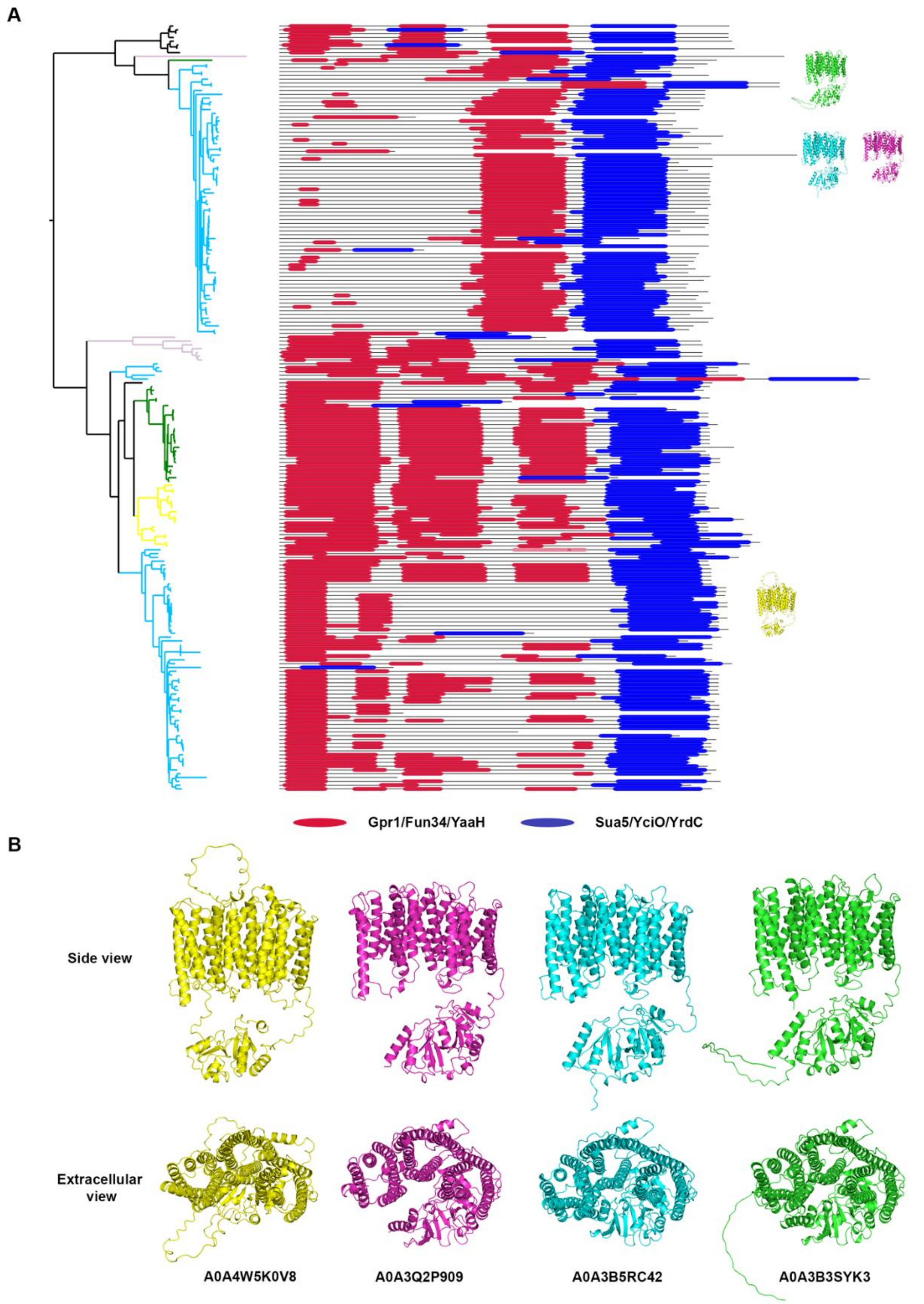

Figure S6. Domain order of vertebrate Atos and representative structures, Related to Figure 7. A. Domain order plotted next to the phylogenetic tree from Figure 6. Domains are plotted on a black line that is proportional to sequence length. Approximate phylogenetic position of the structures shown in B is presented next to the domain plot. **B.** Alphafold 2.0 structures of vertebrate Ato proteins (downloaded from Uniprot) that return less than three hits for the Gpr1/Fun34/YaaH domain. Notice that despite their divergent sequence the three copies of the characteristic six-helix SatP-fold are present in all of them.

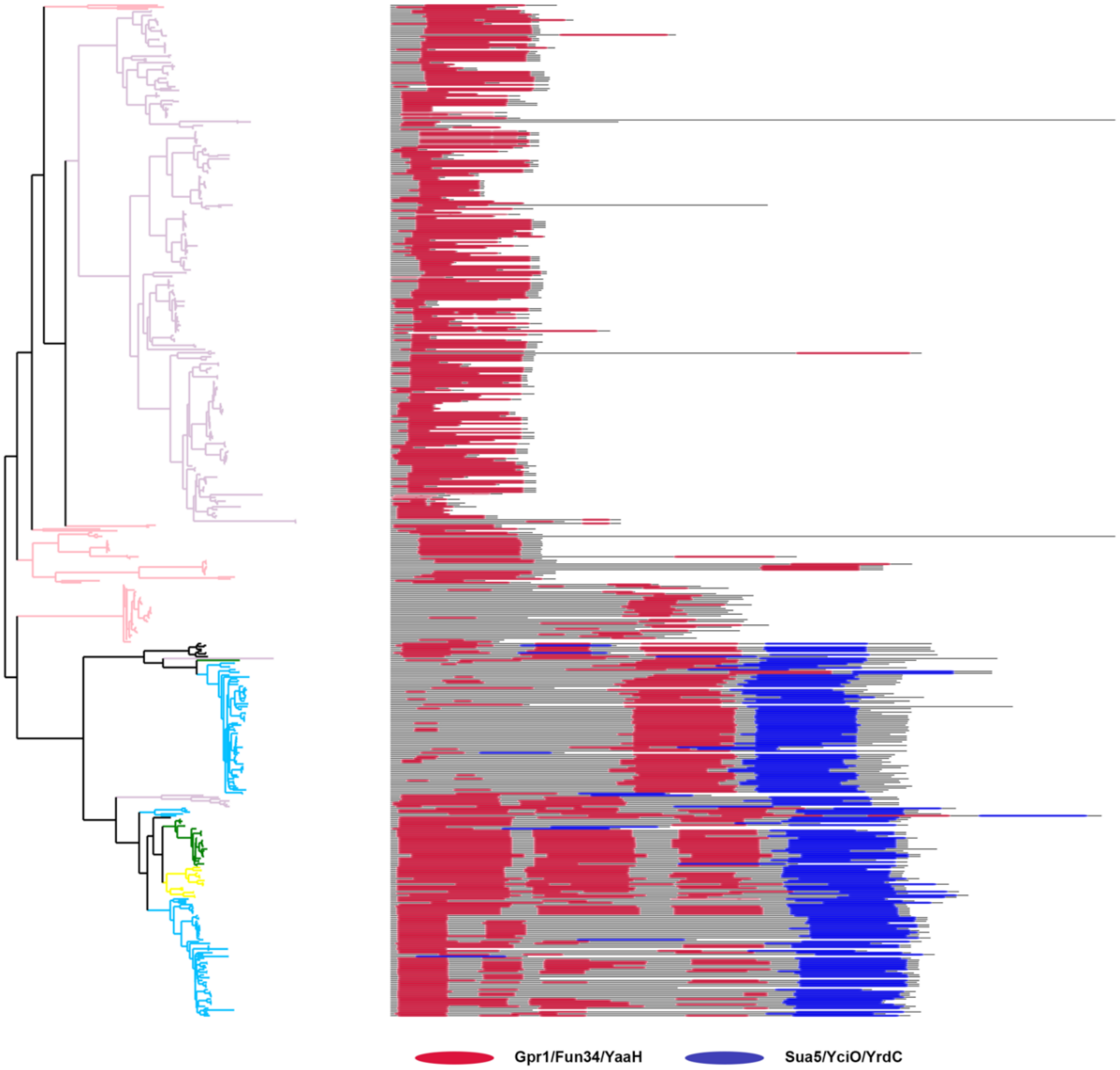

Figure S7. Domain order of metazoan Ato proteins, related to Figure 7. Domains are represented as in Figure S6, and branch colors correspond to those in figure S5. Protostomian sequences are notably shorter, containing a single Gpr1/Fun34/YaaH domain and lacking the C-terminaly fused enzyme-like domain. Their structure is similar to their fungal and prokaryotic homologs.

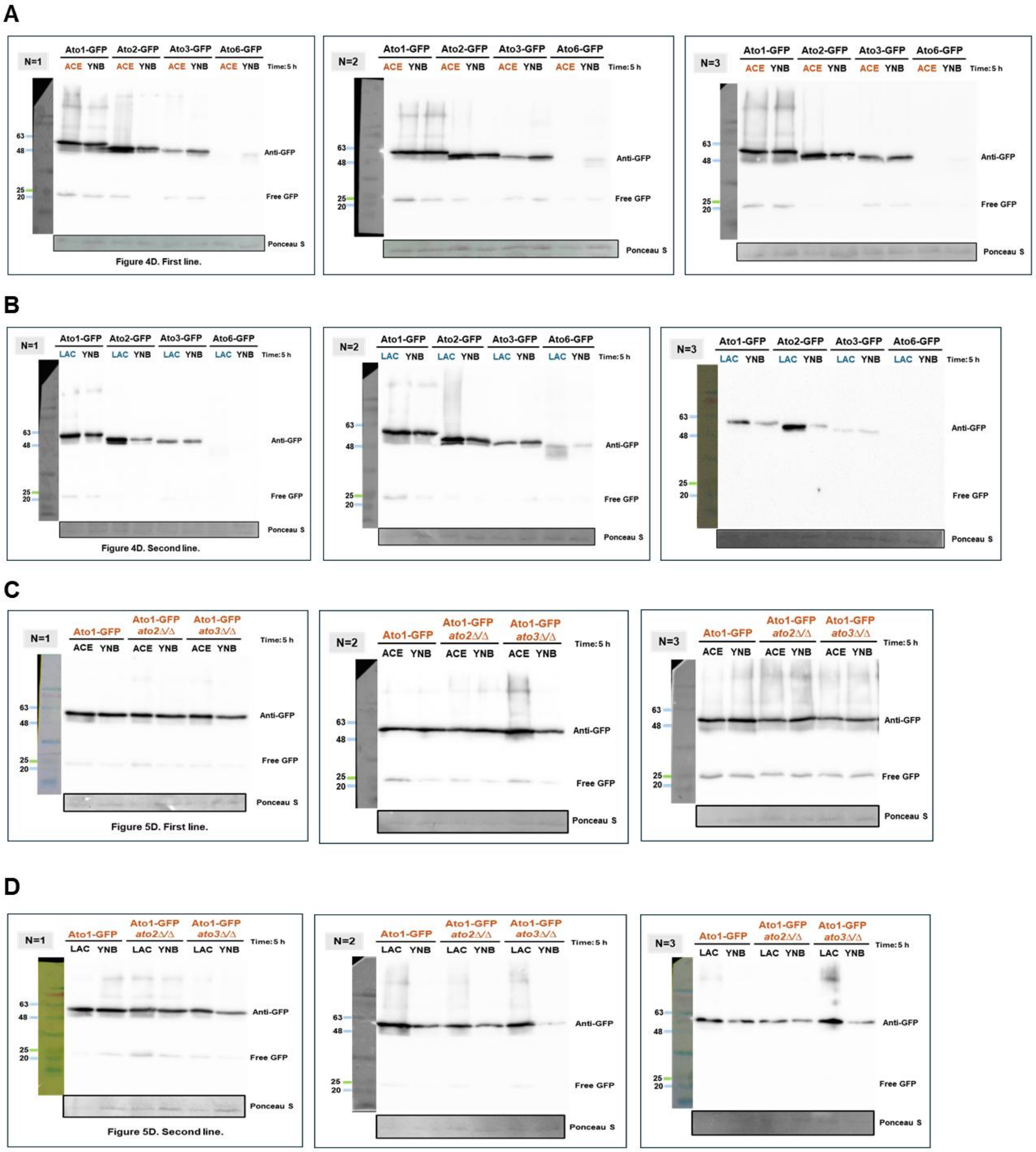
A
N=1
Ato1-GFP Ato2-GFP Ato3-GFP Ato6-GFP
ACE YNB ACE YNB ACE YNB ACE YNB Time: 5 h
63
48
25
20
Anti-GFP
Free GFP
Ponceau S
Figure 4D. First line.
N=2
N=3
B
N=1
Ato1-GFP Ato2-GFP Ato3-GFP Ato6-GFP
LAC YNB LAC YNB LAC YNB LAC YNB Time: 5 h
Anti-GFP
Free GFP
Ponceau S
Figure 4D. Second line.
N=2
N=3
C
N=1
Ato1-GFP
Ato1-GFP ato2Δ/Δ
Ato1-GFP ato3Δ/Δ
ACE YNB ACE YNB ACE YNB Time: 5 h
Anti-GFP
Free GFP
Ponceau S
Figure 5D. First line.
N=2
N=3
D
N=1
Ato1-GFP
Ato1-GFP ato2Δ/Δ
Ato1-GFP ato3Δ/Δ
LAC YNB LAC YNB LAC YNB Time: 5 h
Anti-GFP
Free GFP
Ponceau S
Figure 5D. Second line.
N=2
N=3

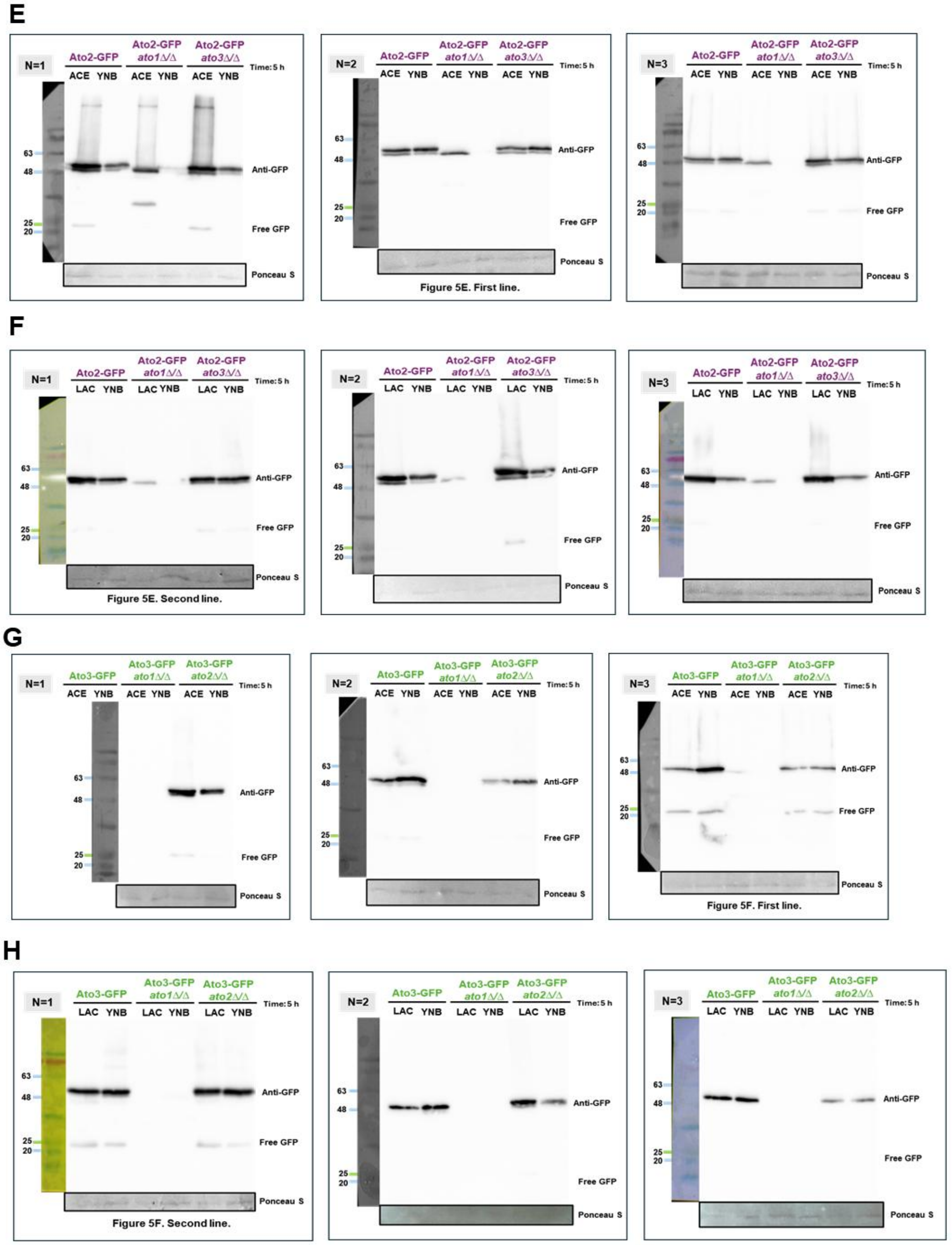

Figure S8. Western Blots of the indicated Ato-GFP fusion proteins in WT and *ato*-mutant strains of *C. albicans,* related to Figure 4 and Figure 5. *C. albicans* cells were grown in 50 mL SM medium supplemented with 0.2% (w/v) glucose to exponential phase. Cells were then washed with deionized water and transferred, to fresh minimal media containing different carbon sources: 0.1% (v/v) acetic acid pH 6.0 (ACE), 0.1% (v/v) lactic acid pH 5.0 (LAC) or no added carbon source (YNB) (SM: 0.67% w/v YNB with ammonium sulphate). **A-H.** Cells were collected at the time-point 5 hours (h), and total protein extracts were separated by SDS-PAGE. Ato-GFP proteins were detected with an anti-GFP antibody. Images show full, uncropped blots and molecular weight markers (kDa) are indicated on the left. Ponceau S staining was used as a loading control. The results represent three independent experiments, each using a different clone (n≥3).

```
# Sequence                  # x   Context     Score   Kinase    Answer
# -------------------------------------------------------------------
# Sequence                  3 S   --MPSTSSQ   0.534   PKC        YES
# Sequence                  4 T   -MPSTSSQK   0.753   PKC        YES
# Sequence                  6 S   PSTSSQKSV   0.921   unsp       YES
# Sequence                  9 S   SSQKSVGSS   0.996   unsp       YES
# Sequence                 13 S   SVGSSVMDP   0.988   unsp       YES
# Sequence                 28 S   KVEVSGDGG   0.958   unsp       YES
# Sequence                 54 T   AFGGTLNPG   0.507   cdc2       YES
# Sequence                 81 T   FALSTFVLS   0.678   PKC        YES
# Sequence                102 S   NIAVSLALF   0.616   PKA        YES
# Sequence                132 T   MTALTSYGA   0.835   unsp       YES
# Sequence                133 S   TALTSYGAF   0.837   PKC        YES
# Sequence                158 S   AYEKSEETV   0.571   CKI        YES
# Sequence                180 T   WAIFTFILW   0.647   PKC        YES
# Sequence                187 T   LWLNTLKST   0.833   unsp       YES
# Sequence                190 S   NTLKSTVAF   0.621   PKC        YES
# Sequence                214 S   AGEFSQKTA   0.744   unsp       YES
# Sequence                230 T   LGVITAIIA   0.502   PKG        YES
# Sequence                245 T   GTATTTNSY   0.593   PKC        YES
# Sequence                248 S   TTTNSYFRP   0.622   PKC        YES
# Sequence                254 S   FRPISIPMP   0.711   PKA        YES
#
    MPSTSSQKSVGSSVMDPNEPPVGKVEVSGDGGEFVVINRHKYYRHELMAA   #    50
    FGGTLNPGAVPWPKININPAPLGLCAFALSTFVLSLFNAQAMGIKIPNIA   #   100
    VSLALFYGGLAQFLAGCWEFVTGNTFGMTALTSYGAFWLSFGAIFIDSFG   #   150
    IVAAYEKSEETVPQLKNALGFYLLAWAIFTFILWLNTLKSTVAFCALFFC   #   200
    LFVTFILLAAGEFSQKTALARAGGVLGVITAIIAWYVALAGTATTTNSYF   #   250
    RPISIPMPGNVAFKN                                      #   300
%1  ..ST.S..S...S..............S......................   #    50
%1  ...T..........................T...................   #   100
%1  .S.............................TS.................   #   150
%1  .......S.....................T......T..S..........   #   200
%1  .............S...............T..............T..S..   #   250
%1  ...S...........
```

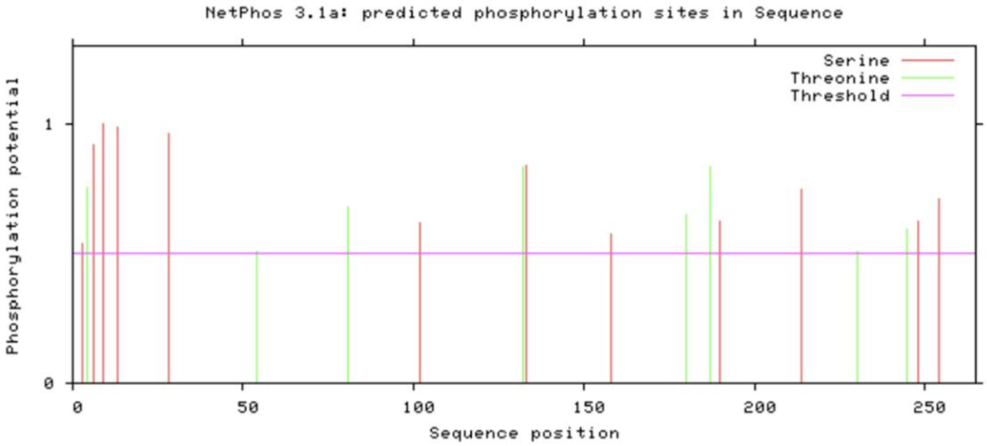

Figure S9. Phosphorylation sites prediction in CaAto2 using Netphos-3.1b, related to Figure 5. A total of 20 putative phosphorylation sites (prediction score ≥ 0.5) were identified, comprising 12 serine (indicated in red) and 8 threonine (indicated in green) residues. Four serine residues at positions 6, 9, 13, and 28 exhibited the highest prediction score (1.000). “#” indicates the position of the residue, and “X” represents the amino acid.

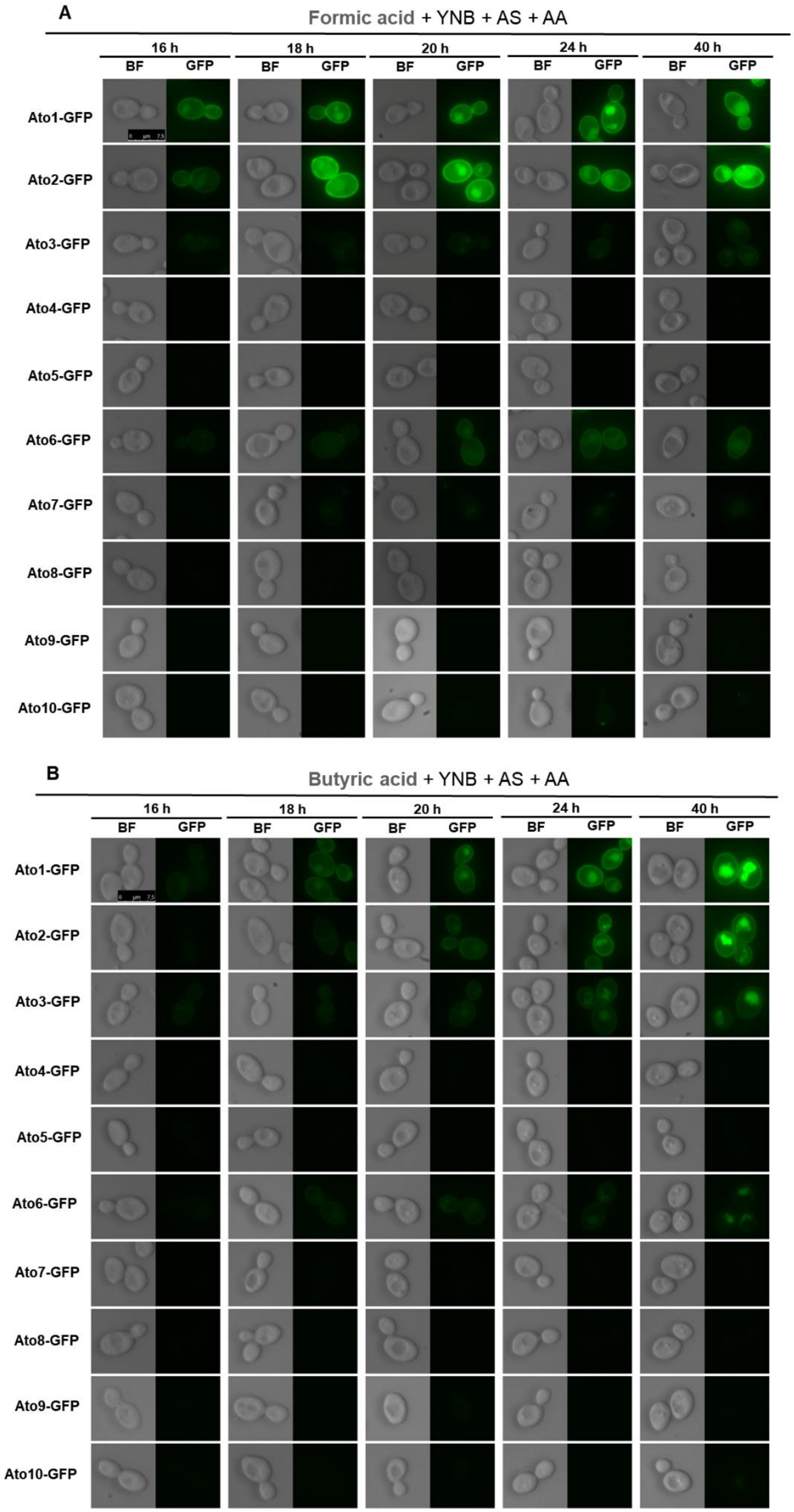
A
Formic acid + YNB + AS + AA
16 h
18 h
20 h
24 h
40 h
BF
GFP
Ato1-GFP
Ato2-GFP
Ato3-GFP
Ato4-GFP
Ato5-GFP
Ato6-GFP
Ato7-GFP
Ato8-GFP
Ato9-GFP
Ato10-GFP
B
Butyric acid + YNB + AS + AA
16 h
18 h
20 h
24 h
40 h
BF
GFP
Ato1-GFP
Ato2-GFP
Ato3-GFP
Ato4-GFP
Ato5-GFP
Ato6-GFP
Ato7-GFP
Ato8-GFP
Ato9-GFP
Ato10-GFP

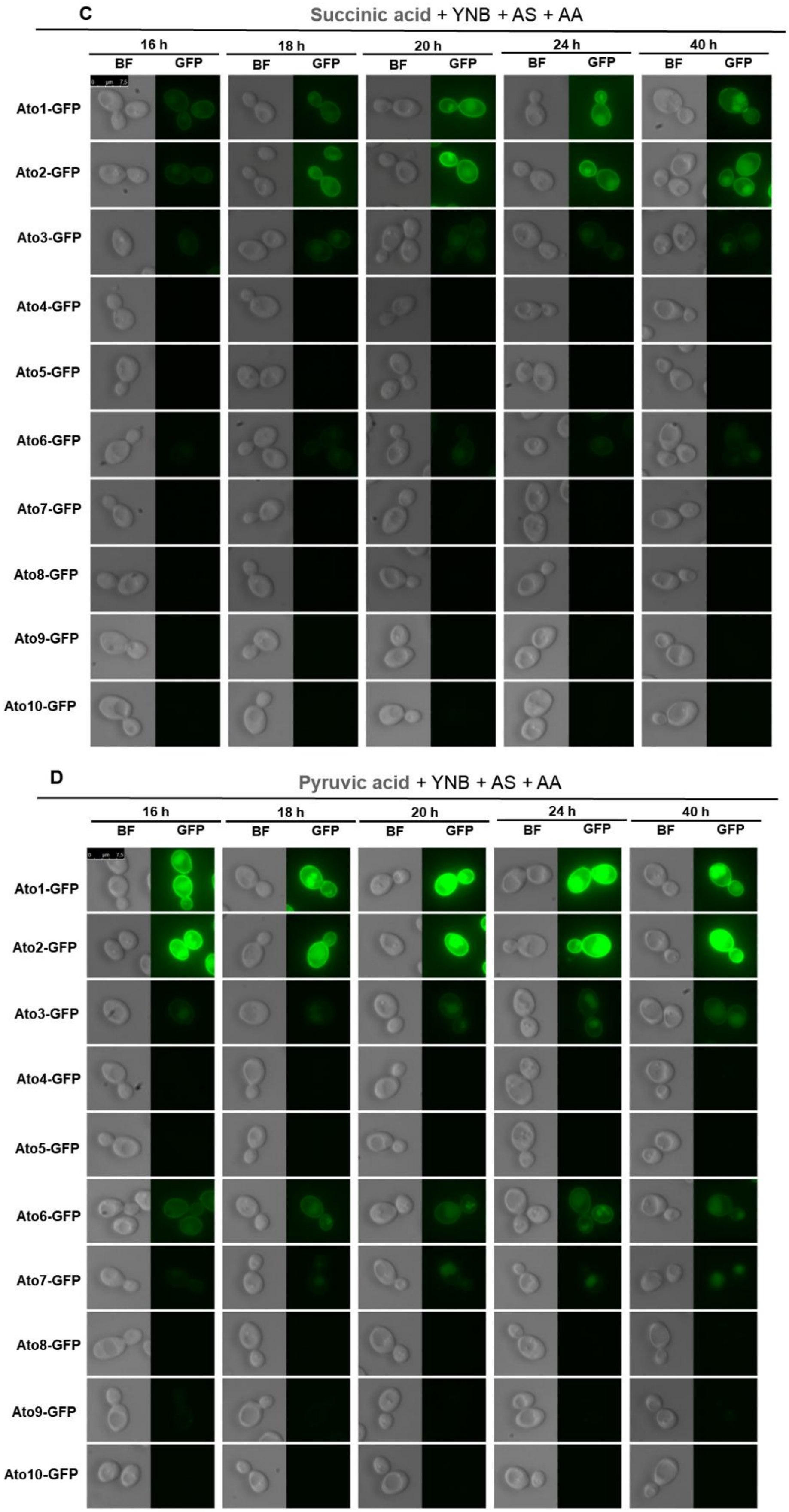
C
Succinic acid + YNB + AS + AA
16 h
18 h
20 h
24 h
40 h
BF
GFP
Ato1-GFP
Ato2-GFP
Ato3-GFP
Ato4-GFP
Ato5-GFP
Ato6-GFP
Ato7-GFP
Ato8-GFP
Ato9-GFP
Ato10-GFP
D
Pyruvic acid + YNB + AS + AA
16 h
18 h
20 h
24 h
40 h
BF
GFP
Ato1-GFP
Ato2-GFP
Ato3-GFP
Ato4-GFP
Ato5-GFP
Ato6-GFP
Ato7-GFP
Ato8-GFP
Ato9-GFP
Ato10-GFP

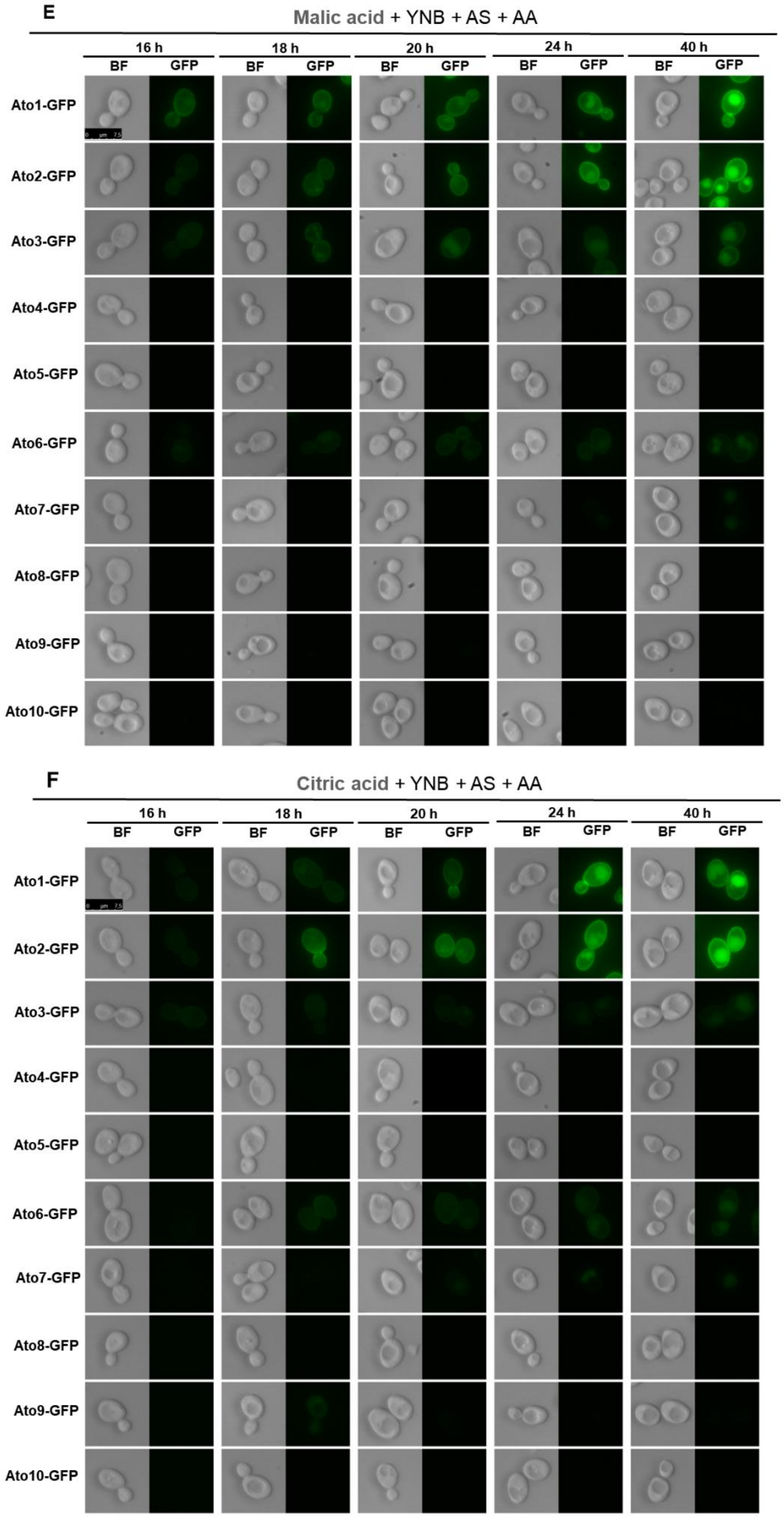
E
Malic acid + YNB + AS + AA
16 h
18 h
20 h
24 h
40 h
BF
GFP
Ato1-GFP
Ato2-GFP
Ato3-GFP
Ato4-GFP
Ato5-GFP
Ato6-GFP
Ato7-GFP
Ato8-GFP
Ato9-GFP
Ato10-GFP
F
Citric acid + YNB + AS + AA
16 h
18 h
20 h
24 h
40 h
BF
GFP
Ato1-GFP
Ato2-GFP
Ato3-GFP
Ato4-GFP
Ato5-GFP
Ato6-GFP
Ato7-GFP
Ato8-GFP
Ato9-GFP
Ato10-GFP

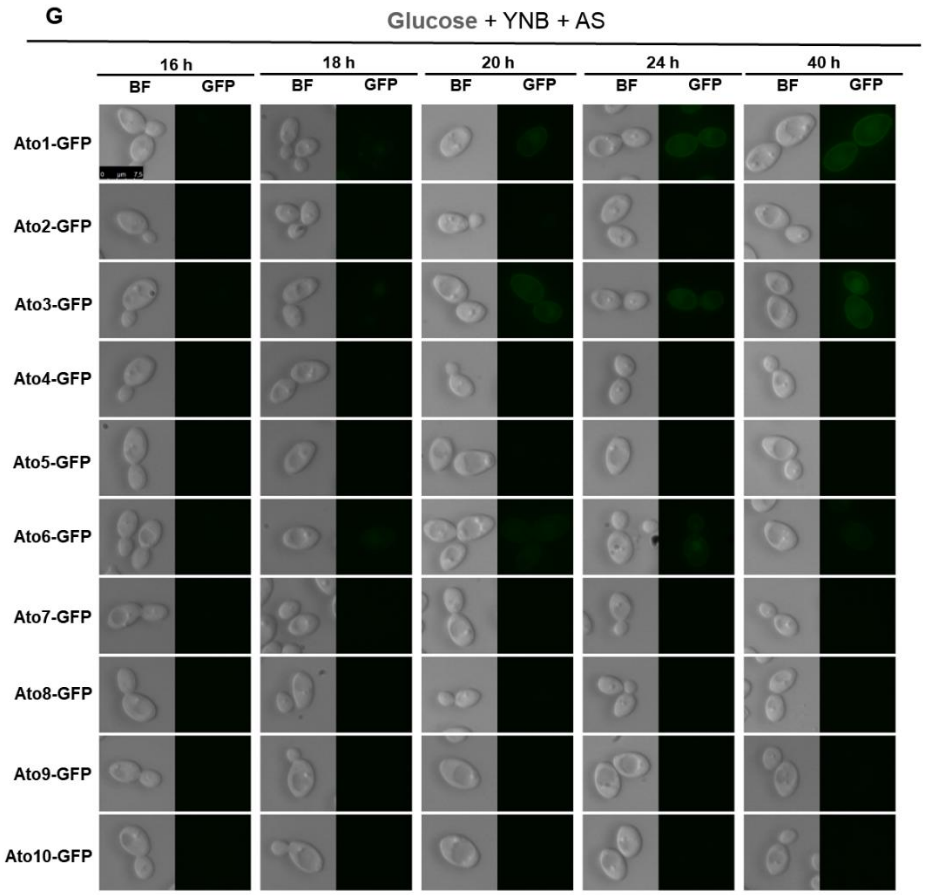

Figure S10. **Subcellular localization of Ato-GFP fusion proteins in live *C. albicans* cells grown on different carbon sources, related to Figure 4.** *C. albicans* cells grown in 20 mL SC medium supplemented with broad range of mono, di or tricarboxylic acids including : **A.** Formic acid (0.1% v/v, pH 5.0), **B.** Butyric acid (0.1% v/v, pH 5.0), **C.** Succinic acid (0.1% w/v, pH 5.0), **D.** Pyruvic acid (0.1% w/v, pH 6.0), **E.** Malic acid (0.1% w/v, pH 6.0), **F.** Citric acid (0.1% w/v, pH 6.0), **G.** Glucose (0.2% w/v) (SM media as control) at 30 °C, 200 rpm. Cells were visualized by fluorescence microscope after 16, 18, 20, 24, and 40 h of direct growth in the specified medium. The time point t≈16 h corresponded to the beginning of exponential phase (0.4 < $OD_{640\ nm}$ < 0.6). Scale bars, 7.5 µm. The abbreviations BF and GFP refer to "Bright-Field" and "Green Fluorescent Protein," respectively.

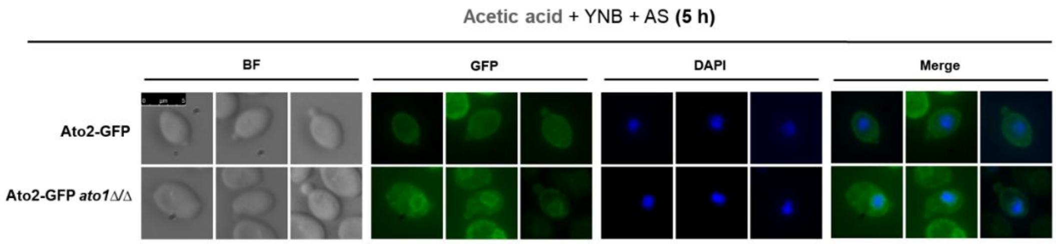

Figure S11. Subcellular localization of Ato2-GFP in *C. albicans* Ato2-GFP and Ato2-GFP *ato1Δ/Δ* cells, related to Figure 5. Cells were grown in 50 mL synthetic minimal (SM) medium supplemented with 0.2% (w/v) glucose to exponential phase, washed with deionized water, and transferred to fresh minimal medium containing 0.1% (v/v) acetic acid (pH 6.0) for induction. After 5 h, cells were harvested, fixed with 3.7% PFA, and stained with DAPI (2 μM, 10 min) to visualize nuclei. Scale bar, 5 μm. The abbreviations BF, GFP, DAPI, and Merge refer to "Bright-Field", "Green Fluorescent Protein", "4′,6-diamidino-2-phenylindole", and "the merged image of GFP and DAPI channels", respectively.

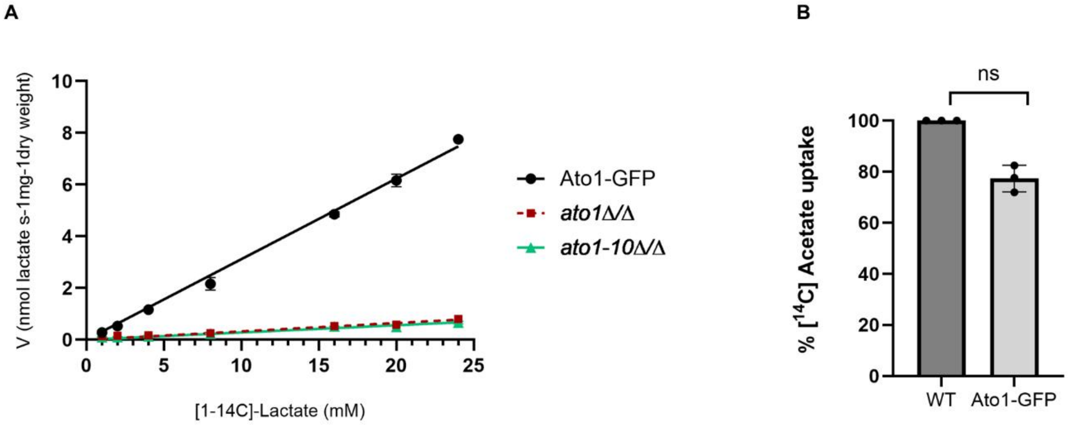

Figure S12. Uptake of lactate and acetate in *C. albicans* WT, Ato1-GFP, *ato1Δ/Δ* and *ato1-10Δ/Δ* cells, related to Figure 6. *C. albicans* cells were harvested after 5 h of derepression in synthetic minimal medium containing 0.1% (v/v) acetic acid (pH 6.0). **A.** Uptake of [^{14}C]lactate ranging from 1 to 24 mM in Ato1-GFP, *ato1Δ/Δ* and *ato1-10Δ/Δ* cells. **B.** Transport of radiolabeled [^{14}C]acetate (4 mM, pH 6.0) in WT and Ato1-GFP cells. Data was tested for normality prior to analysis. Statistical analysis: Mann–Whitney U test**.** ns, not significant.
